# Dosage effect of copy number variation in epilepsy and ten regions of the human brain

**DOI:** 10.1038/s41598-025-28338-2

**Published:** 2025-12-04

**Authors:** Tisham De, Lachlan Coin, Michael R. Johnson

**Affiliations:** 1 Department of Epidemiology and Biostatistics, School of Public Health, Imperial College, London, UK; 2 Department of Genomics of Common Diseases, Imperial College, London, UK; 3 Department of Infectious Disease, Imperial College, London, UK; 4 Department of Microbiology and Immunology, University of Melbourne at The Peter Doherty Institute for Infection and Immunity, Melbourne, Australia; 5 Department of Brain Sciences, Imperial College, London, UK

**Keywords:** Genetics, Neuroscience

## Abstract

Epilepsy and seizures are common neurological conditions which often manifest with complex symptoms. Several studies including large scale genome-wide association studies (GWASs) and exome studies have reported several catalogues of genes related to epilepsies and seizure generation. Similarly, there exists several successful studies elucidating the role of single-nucleotide polymorphisms (SNPs) in expression quantitative trait loci (eQTLs) for the human brain. Here, as one of few studies in the current literature we have analysed and reported the dosage effect of small-to-intermediate length copy-number variants (CNVs) in two epilepsy cohorts characterised for phenotypes such as seizure counts, seizure frequency and remission to anti-epileptic drugs (AEDs). In addition, we have performed comprehensive CNV based eQTL analysis in different regions of the normal human brain from the United Kingdom Brain Expression Consortium (UKBEC) study. We leveraged all analyses to decipher new genes for epilepsy phenotypes such as seizure frequency and further uncovered genetic controls of neurotransmitters like serotonin, dopamine and signalling molecules like G protein-coupled receptors (GPCRs). Importantly, we observed and have reported clustering of *cis* CNV-QTL signals in specific regions of the human genome such as the chromosome 1p36 proband containing the GNB1 gene or the chromosome 9q22 proband containing NANS. This observed phenomenon of clustering or co-localisation of association signals was further corroborated by our non-negative matrix factorization (NMF) analysis of the UKBEC gene expression data. To conclude, our results here successfully describe in detail the dosage effect of CNVs for epileptic seizures and further elucidates its role in the genomic architecture of gene expression in various regions of the human brain.

**Supplementary Information:**

The online version contains supplementary material available at 10.1038/s41598-025-28338-2.

## Introduction

Epilepsy is a common neurological disease affecting around 1% of the population worldwide. Anti-epileptic drugs (AEDs) in general work well for 60% of epilepsy patients who successfully achieve seizure control with current medication within a year or two, however for around one third or 20-30% patients the latest AEDs (25 licensed drugs worldwide^1,2^) do not work well and these patients continue to have regular seizures^3,4^. Further, it has been observed that resistance to one AED correlates well with resistance to all other drugs. One off seizure is quite common in young children and adults but usually termed benign. For some refractory groups of epilepsy patients who do not respond to standard medication a surgery may be required to control seizures^5,6^. In some rare cases of epilepsy known as Lennox-Gastaut syndrome (LGS), which often originates in the occipital lobe of the brain, a child may have numerous seizures in a day. As a part of the CADET (Children’s Adaptive Deep brain stimulation for Epilepsy Trial) trial, United Kingdom’s first patient, a child with LGS with mutations in the SCN1B gene was successfully implanted with a device in the brain to control seizures through electric pulses. Chromodomain-helicase-DNA-binding protein 2 or the CHD2 gene is another candidate gene for this syndrome^7^.

However, little is known about the biology of brain seizures, the regions where it originates and the causal mechanisms behind it. Thus, the genetic basis of seizures remains an open question in neurology. Here, we present comprehensive copy-number variation (CNV) analyses for epilepsy phenotypes including seizure counts, seizure frequency and 12-month remission to AEDs in two cohorts denoted as SANAD (the Standard and New AED clinical trial)^8^ and Australian cohort^4^. In addition, we have also analysed and reported here CNV gene expression signatures (CNV-eQTLs) in different regions of the normal human brain from the United Kingdom Brain Expression Consortium (UKBEC)^9^ and the North American Brain Expression Consortium (NABEC)^10–12^ studies. We leveraged all analyses to decipher new gene clusters and loci for the neurobiology of epileptic seizures and report the phenomenon of reciprocal CNV dosage in genes related to neurotransmitters and G protein-coupled receptors (GPCRs) mediated signal transduction in different regions of the human brain.

## Results

### CNV analysis in epilepsy cohorts

**Fig. 1:**
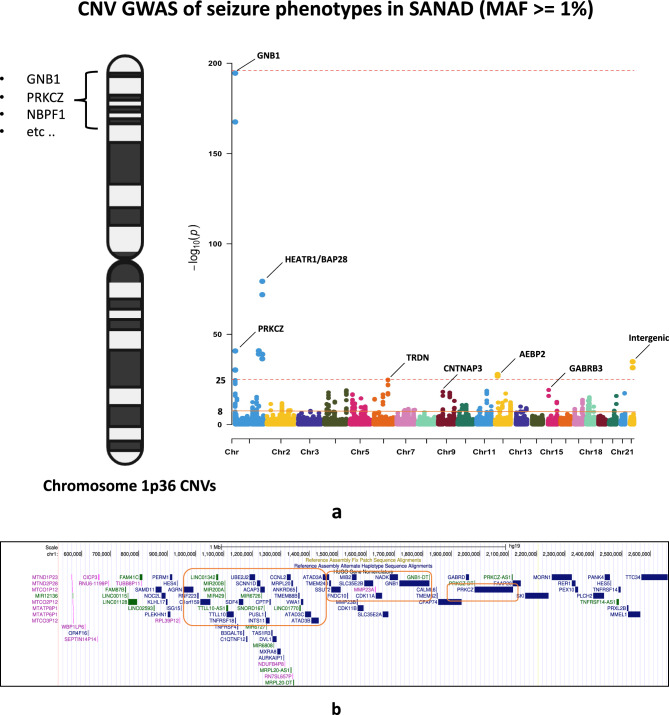

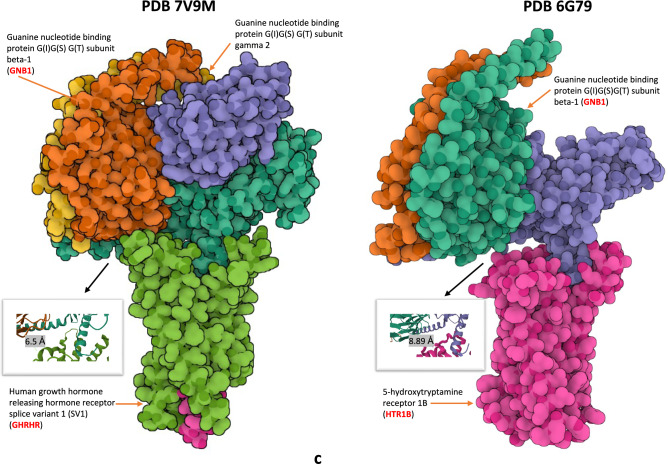
GNB1 in SANAD (**a**) Manhattan plot showing CNVs (including GNB1) associated with seizure related phenotypes in the SANAD cohort. Genes with P value < 1x10^-25^ are highlighted and marked. (**b**) GNB1 gene cluster. Figure showing the ~ 1 megabase gene cluster in the chromosome 1p36 region for CNVs significantly associated with seizure related phenotypes in SANAD. Notable results included TTLL10, GNB1 and PRKCZ. (**c**) Protein structures of GNB1. Three-dimensional protein structures from the pdb database showing the protein complexes of GNB1 bound to (i) growth hormone receptor protein GHRHR^67^ (10.2210/pdb7V9M/pdb) and (ii) serotonin receptor protein HTR1B^68^ (10.2210/pdb6G79/pdb). Figures were generated through the Mol* software^66^ (see code section)

In our main discovery cohort SANAD, GNB1 was the top signal for univariate CNV-genotype association model using MultiPhen^13^ (MultiPhen: A Package to Test for Multi-Trait Association- version 2.1.8, https://github.com/DE-Tisham/MultiPhenv2.1.8, see code section) for the phenotypes (1) total number of seizures (chr1:1,745,726 (GRCh37); *P* = 2.89x10^-168^; minor allele frequency (MAF) =1.1%) and (2) seizure frequency (chr1:1,745,726; *P* = 2.82x10^-195^; MAF=1.1%) (Fig. 1a**, **Supplementary Table 1**a**). Next, in the multivariate analysis for CNV genotypes, GNB1 replicated as the top hit in the MultiPhen joint model (see methods) at chr1:1,745,726 with *P* = 6.3x10^-202^ and again in the MultiPhen joint model with variable selection at chr1:1,745,726 with *P* = 2.27x10^-207^ (Supplementary Table 1**a**). In the ClinVar database^14^ annotations for GNB1, we observed that pathogenic or likely pathogenic CNVs (140/184 records) were more numerous than pathogenic SNVs (27/293 records) (Supplementary Table 2). These observations add weight to the hypothesis that CNVs are likely to be functionally relevant in the chromosome 1p36 region. In Log-R ratio (LRR) based association models, the strongest signal was found within growth hormone receptor gene (GHR) (chr5:42,569,642; *P* < 6x10^-128^; Supplementary Table 1**a**). In SANAD, the top gene for drug-response was TRAPPC9 (chr8:140,765,991; *P*= 1.7x10^-05^, LRR univariate model; Supplementary Table 1**a**). TRAPPC9 is used for the clinical diagnosis of a rare neuro-endocrine disease known as Intellectual disability-obesity-brain malformations-facial dysmorphism syndrome^15^ (https://www.malacards.org/).

We further noticed that in SANAD, GNB1 along with several other seizure-associated genes like PRKCZ (chr1:2,082,566; *P* = 1.8x10^-41^; MAF=1.08%) and CDK11A (chr1:1,645,366; *P* = 6.25x10^-8^; MAF=2.4%) were clustered within a one megabase window in the chromosome 1p36 region (Fig. 1b). Of note, GNB1 has been shown to bind with human growth hormone-releasing hormone receptor (GHRHR) and 5-hydroxytryptamine receptor 1B (HTR1B or serotonin receptor) (Fig. 1c), thus indicating its relevance in important brain functions. We also note that the chromosome 1p36 region is relevant for seizures since it is used for karyotyping and clinical diagnosis of 1p36 deletion syndrome^16,17^*.* This observation further corroborates our results in SANAD where for seizure-related phenotypes we found numerous contiguous probes with MAF >1% and significantly low P values (e.g., NBPF1; Supplementary Table 3).

In SANAD, outside the chromosome 1p36 region we found and have reported several genes of interest including six genomic loci exceeding the threshold of P < 1x10^-25^ and MAF >=1% (Fig. 1a). Amongst these loci some notable genes of interest for neurology included HEATR1, CNTNAP3 and GABRB3 (see discussion section for further details). In the independent analysis of the Australian cohort, on using the MultiPhen univariate model for CNV genotypes, the top gene of interest for 12-month remission to AEDs was found to be in the PPFIA2 gene (chr12:82,081,470; P = 6.21x10^-06^; MAF= 4.4%). PPFIA2 also replicated as the top hit in the CNV-genotype multivariate joint model with variable selection (chr12:82,081,470; *P* = 2.21x10^-06^; Supplementary Table 1**b**). PPFIA2 belongs to the LAR (Leukocyte common antigen-related^18^) protein-tyrosine phosphatase-interacting protein (liprin) family^19^ and is known to be involved in pathways related to neurotransmitter release cycle and transmission across chemical synapses (https://pathcards.genecards.org/). Previous studies^20^ have indicated that Ca^2+^ modulated lirpin-α proteins capture KIF1A-driven dense core vesicles (DCV) in dendritic spines^20^.

In the MultiPhen joint meta-analysis of SANAD and Australian cohorts the top gene of interest was DAGLA (chr11:61,462,424; *P* = 0.000301; MAF=1%; Supplementary Table 1**c**). DAGLA is a neural stem-cell derived dendrite regulator which is involved in 2-Arachidonoylglycerol (2-AG) signalling in the central nervous system (CNS)^21–23^. It helps in axonal growth during neurogenesis in early stages of development and in addition helps with neuroinflammatory response in the brain. Other genes of interest in the meta-analysis results included ASIC2 (Acid Sensing Ion Channel Subunit 2; chr17:31,454,867; *P* = 8.54x10^-19^) which was the top hit in LRR multivariate models. Lastly, GNB1 successfully replicated in the LRR univariate meta-analysis model at chr1:1,793,111 with meta-P = 9.49x10^-16^.

Across all analyses and cohorts, the most distinct common CNV-phenotype signal with the highest number of contiguous probes and significant P values was found within the WWOX gene (MAF=47%, 48 contiguous probes for CNV genotypes and 66 contiguous probes in the LRR analysis; Supplementary Table 4). This CNV was found to be exclusively associated with the drug-response phenotype in SANAD. Though WWOX is a well-known candidate gene for epilepsy and could potentially have real seizure related effects in the brain^24–26^, its association with the drug-response phenotype through CNV genotypes in the Australian cohort was less convincing (since it had no CNVs with MAF > 1%). However, in the meta-analysis of CNV genotypes strongest meta-P value was 0.00011 at chr16:79,246,323 for the phenotype ​​Time to 12-month remission and in the LRR based meta-analysis the meta-P value was 4.65x10^-22^ at chr16:79,043,240 for the phenotype Time to first seizure censoring variable (see data section for all results). Since WWOX has a well-known fragile site (FRA16D^27^) further experimental validation is required to confirm these findings.

### Functional validation of GNB1-seizure associated region

**Fig. 2:**
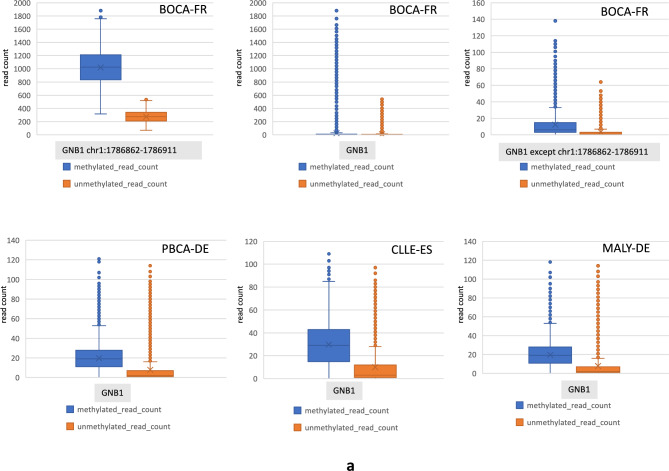

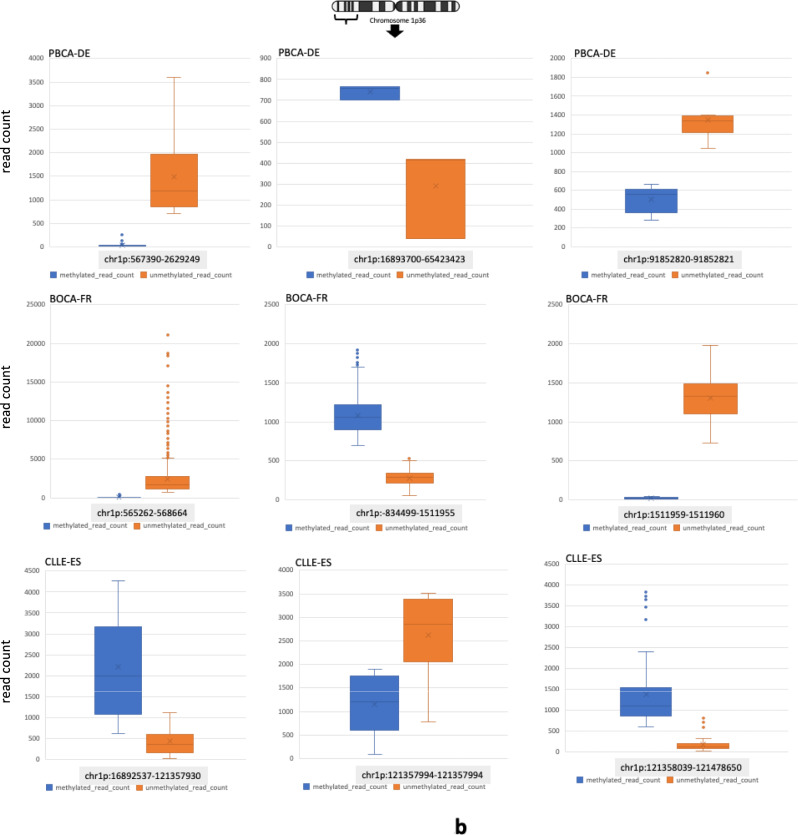

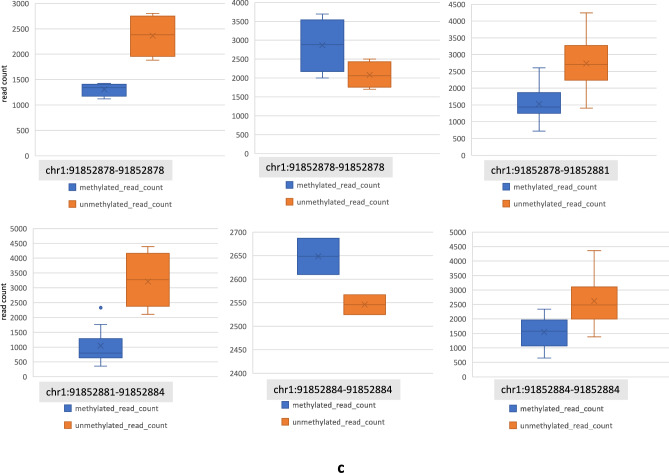
Bisulfite-seq methylation data for GNB1. (**a**) Plot showing raw methylation data from whole-genome bisulfite sequencing for GNB1 in four cancer cohorts. Maximum methylation was found in the region chr1:1786862-1786911 (GRCh37) in Ewing sarcoma cancer cohort from France (BOCA-FR, see methods). This region lies within the GNB1-seizure associated locus in SANAD. (**b**) Figure demonstrating methylation waves in the chromosome 1p36 region in different cancer cohorts. Here, the average read count was > 700 in all cohorts. (**c**) Methylation waves in the HFM1 gene. See Supplementary Table 6 for HFM1 data.

Briefly, in SANAD the GNB1-seizure associated region of interest (chr1:1,745,726-1,810,090; Supplementary Table 3) is an intronic region that spans approximately 64 kilobase pairs with six unique genotyping probes available for analyses. We found that all six GNB1 probes had extreme P values for the two seizure related phenotypes in SANAD namely seizure frequency and total number of seizures (Supplementary Table 3). Next, we aimed at functional validation of this seizure associated region in GNB1. For this, we first highlight that GNB1 lies within the distal critical region for 1p36 deletion syndrome. This region has been clinically tested and experimentally validated by numerous studies and has been reviewed in Fig. 3 of Jordan et al. 2015^17^. Next, to further test the hypothesis of possible epigenetic activity in the chromosome 1p36 region or in GNB1, we analysed whole-genome bisulfite-seq methylation data in four different cancer types, including for (a) paediatric brain cancer (PBCA-DE) and (b) soft tissue cancer - Ewing sarcoma (BOCA-FR) (see methods). Interestingly, in BOCA-FR, we identified a highly methylated region of interest at chr1:1,786,862-1,786,911 (GRCh37) (which lies within the GNB1-seizure associated locus in SANAD or chr1:1,745,726-1,810,090) where the average methylation read count values were 50x higher than GNB1 (whole gene) methylation data in other cohorts. For instance, in PBCA-DE the average methylated read count for whole of GNB1 region was found to be ~20 compared to the average methylated read count of >1,000 in the chr1:1,786,862-1,786,911 region of GNB1 in BOCA-FR (Fig. 2a). We believe that such high levels of methylation observed in the GNB1-seizure associated locus possibly indicates epigenetically driven regulation of GNB1, but also note that its relevance for neurological phenotypes in epilepsy (like seizures) currently remains unknown. These epigenetic results were further strengthened by our RNA-seq analysis of BOCA-FR where the absolute read count for GNB1 RNA was approximately 10x higher than the expression of other epilepsy genes of interest like WWOX (Supplementary Fig. 1**a**)

### Epigenetic waves in the chromosome 1p36 region

The unusually high level of methylation in GNB1 in BOCA-FR led us to question the relevance of these results on a genome-wide scale. In the four cancer cohorts we analysed we discovered that the chromosome 1p36 region was consistently amongst the top regions in the genome where the average methylation levels are higher by at least a factor of 10. Apart from the 1p36 region some other examples of regions with such high methylation activity (with mean methylated read counts >700) were found to be located near the centromeres, the Y chromosome and several other genes of interest like HFM1, GEN1 (involved in homologous recombination and double-strand break repair through Holliday junction) and PMF1 (Polyamine Modulated Factor 1) (Supplementary Table 5). Importantly, here we report a new epigenetic phenomenon where we observed the methylation levels in the genome to fluctuate in a linear and periodic manner. We refer to these fluctuations as *methylation waves*. We observed that such epigenetic waves tend to occur at a higher amplitude in the chromosome 1p36 region (compared to other genes like WWOX) and interestingly, can also flip in the opposite direction in a group of individuals at the same genomic location (Fig. 2b, c; Supplementary Table 6). A notable example of this phenomenon, observed only in the paediatric brain cancer cohort (PBCA-DE) was in the HFM1 gene (Helicase for Meiosis 1) (Fig. 2c; Supplementary Table 6). HFM1 is known for its role in DNA double-strand break repair, crossover formation and complete synapsis of homologous chromosomes and regulation of the BRCA1 gene^28^. Further, on analysing genome-wide CNV results in all our cancer cohorts we discovered that compared to other cohorts PBCA-DE had the least number of CNV calls (lower by several orders of magnitude) and no CNVs segments detected on the chromosome 1p36 proband or chromosome 1 (Supplementary Table 7). These results indicate that in paediatric brain cancers (PBCA-DE) high methylation activity in the chromosome 1p36 region or in genes like HFM1 (average methylated read count = 1,448; average unmethylated read count= 2,493) might confer genome stability (e.g. through BRCA1 interaction)^28^ or may even suppress the formation of CNVs on chromosome 1. Of note, the average methylated read counts in the intronic deletion region in WWOX (chr16:78,371,638-78,385,000) was found to be 6.8 in BOCA-FR, 20.57 in PBCA-DE and 31.6 in CLLE-ES. After excluding data for the deletion region, the mean methylated read counts for the WWOX gene was found to be 7.9 in BOCA-FR, 23.75 in PBCA-DE and 36.15 in CLLE-ES (Supplementary Fig. 1**b**; Supplementary Table 8).

### CNV dosage effects in the UKBEC and NABEC cohorts

**Fig. 3:**
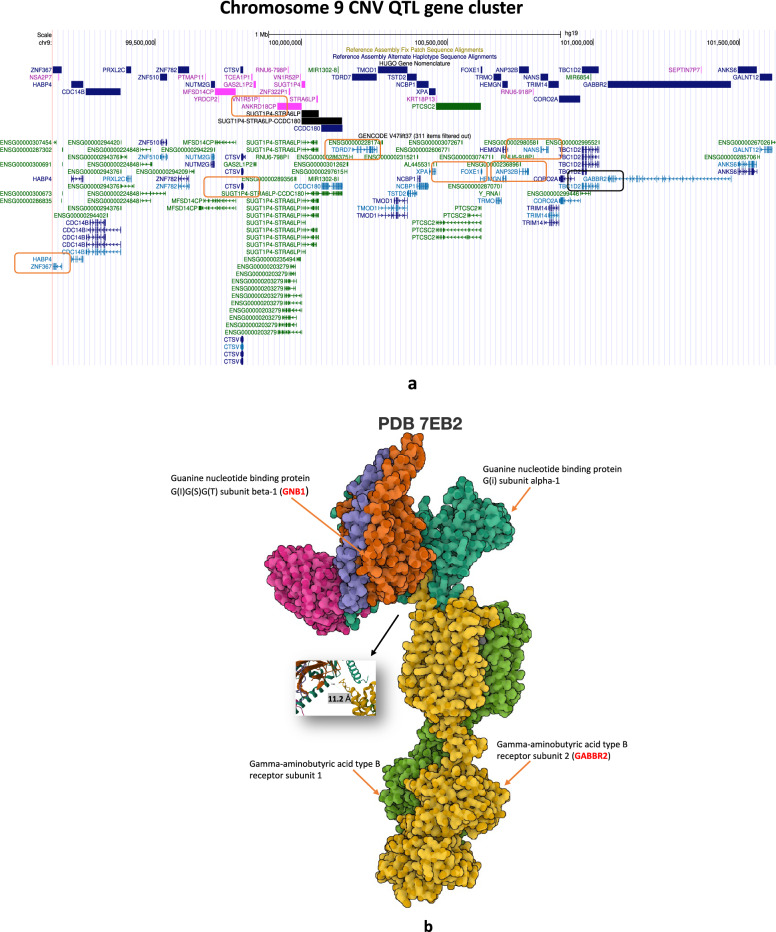
CNV-QTL results in the UKBEC study. (**a**) Figure highlights the ~ 1 megabase window where the top (rank 1) hits from UKBEC analysis for ten brain regions were found to be clustered (Supplementary Table 1 d,e; GRCh37). Notable genes included TDRD7, ANP32B and NANS (GABBR2 also lies within this cluster and is a known epilepsy gene but was not found to be significant in our results). (**b**) Protein complex from the pdb database depicting the three-dimensional structure of GNB1 bound to GABBR2^69^ (10.2210/pdb7EB2/pdb). Figure generated through the Mol* software^66^ (see code section)

**Fig. 4:**
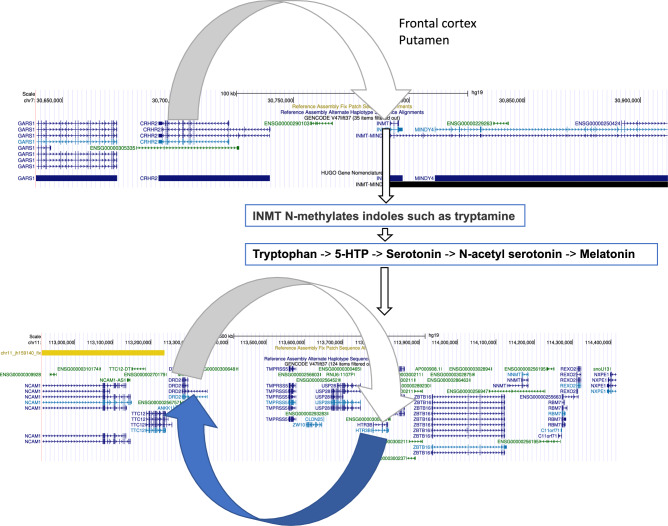
The genetic link between cortisol, serotonin and dopamine. The top panel shows the *cis* CNV-QTL of cortisol receptor gene CRHR2 with INMT and its link to the tryptamine metabolite. The bottom panel shows a reciprocal *cis* CNV-QTL (or dosage) of DRD2 and HTR3B and its link to the serotonin synthesis pathway.

Briefly, in the UKBEC study we generated two sets of CNV calls from two different genotyping platforms namely Illumina Infinium Omni1-Quad BeadChip array and a custom Illumina Immunochip^9^. Next, we analysed these two datasets independently with Illumina platform specific emission parameters in cnvHap^29^. In the Omnichip dataset we detected 9,242 homozygous deletions (type 0), 129,929 heterozygous deletions (type 1), 7,840 heterozygous duplications (type 3) and 546 homozygous duplications (type 4). Genome-wide CNV breakpoint information for all cohorts is available in the data section. Next, for every probe in all cohorts we derived expected CNV genotypes based on posterior probability in cnvHap (see methods). After this, using the MultiPhen method^13^ (which consists of both univariate and multivariate approaches) we associated CNV genotypes with gene expression values from different brain regions in the UKBEC and NABEC datasets (see methods). In total, for the UKBEC analyses we generated 96 sets of transcriptome-wide CNV-QTL results spanning CNV-genotypes, LRR, Multiphen univariate and MultiPhen multivariate methods. The top rank 1 results for these analyses is reported in Supplementary Table 1**d, e.** The most notable observation in these results is a cluster of significant CNV-QTLs within a one mega base pairs window on chromosome 9 (GRCh37), which consistently harboured the top hit in different brain regions. Two genes of interest in this cluster included TDRD7 (*P* < 1x10^-269^; MAF=2.6%, for CNV genotypes) and NANS (*P* < 1x10^-269^; LRR based models) (Fig. 3a; Supplementary Table 1**d**). N-acetyl-neuraminic acid synthase or the NANS gene synthesises sialic acid in humans and has the highest concentration in the brain. Bi-allelic recessive mutations in NANS are known to cause intellectual disability with short stature^30^ and plays an important function in neural transmission and ganglioside structures in synaptogenesis^31^. In the UKBEC Immunochip results, the top hit was ANP32B (P value < 1x10^-269^; MAF=1.8%) which also happens to be located within the chromosome 9 gene cluster containing TDRD7 and NANS. Here, ANP32B, like TDRD7 and NANS, was consistently the top hit in all eight brain regions (Supplementary Table 1**e**). ANP32B is known to regulate gene expression^32^ and leads to transcriptional repression of the KLF5 gene^33^. Interestingly, NANS and ANP32B also lie near GABBR2. GABBR2, though not associated with any of our epilepsy phenotypes or had any CNV with MAF >1% in any of our cohorts, is known to cause Developmental and epileptic encephalopathy 59 (DEE59)^34^ (https://www.malacards.org/). Separately, it was also shown to bind with GNB1 in a protein receptor complex (Fig. 3b). Further, to complement the RNA side of protein interaction data for GNB1 (e.g., with HTR1B; Fig. 1c), results from the UKBEC Omnichip dataset indicated hippocampus to be the top brain region of interest (strongest P values) for transcriptional co-regulation (Supplementary Figs. 2-5). Brain-region-specific CNV-QTL results (heatmaps) for other seizure associated epilepsy genes (e.g., WWOX) and chromosome 9q22 cluster genes (e.g., NANS and GABBR2) are provided in Supplementary Figs. 6-14. In our analyses of the UKBEC and NABEC datasets we discovered many additional significant loci and regions of interest. One important example we highlight here is the significant *cis* CNV-QTL between serotonin and dopamine receptors on chromosome 11. In this instance, we found probes in the HTR3B gene to be significantly associated with DRD2 gene expression values (e.g. CNV genotypes at chr11:113,802,601 with MAF=1.1% associated with DRD2 probe id 3391654 with P = 5.88x10^-81^ in the white matter brain region) and conversely probes in the DRD2 gene was found to be significantly associated with HTR3B gene expression (e.g. LRR in the DRD2 gene was associated with HTR3B probe id 3349661 with P < 1x10^-308^ using LRR MultiPhen joint model with variable selection method in in cerebellum brain region; Fig. 4**;** see data section for complete set of results). Additional significant association signals were also observed in multiple loci for these two genes and in other related genes (see data section for complete set of results). Interestingly, further delving into the serotonin biosynthesis pathway, we additionally discovered significant *cis* CNV-QTL for the cortisol receptor gene CRHR2. CRHR2 was found to be associated with the expression of the nearby INMT gene (P < 1x10^-20^) in putamen and frontal cortex brain regions through the LRR MultiPhen joint model with variable selection. INMT is known to N-methylate indoles such as tryptamine, which interacts with tryptophan to produce serotonin (Fig. 4).

So far, all our analyses are based on an overlapping moving window across the genome i.e. CNV calling and association were performed for segments of the genome consisting of both genic and intergenic regions (see methods). In addition to this, for the UKBEC Omnichip dataset, we also performed CNV analysis on a gene-by-gene basis. The motivation behind this approach is to find CNVs lying within a gene and to further uncover its local effect on exonic expression. This approach could potentially help us identify the relative importance of exons in a given tissue. To this end, we called CNVs through the cnvHap HMM model, but for all human genes individually (with a 5-kilo base pair window around every gene boundary). Next, like before, we derived expected CNV genotypes and associated them with eight different UKBEC gene expression matrices using the MultiPhen univariate and multivariate association models. Some notable examples of genes with common CNVs are as follows. For the putamen brain region and in the HEATR1 gene the CNV-dosage analysis using MultiPhen joint model with variable selection identified exon 4 to be most significant (probe id=2462530), for PRKCZ it was exon 10 (probe id=2316299) and for WWOX it was exon 5 (probe id=3700865) (Supplementary Fig. 15). A joint consensus analysis of important exons identified through CNV genotypes, LRR, MultiPhen univariate and multivariate methods remains to be explored. Lastly, in the CNV-QTL results for the NABEC cohort (Supplementary Table 1**f**) the top two genes common to both cerebellar cortex and frontal cortex were (1) TFG (e.g., in cerebellar cortex chr3:100,424,728; P value=2.96x10^-83^) and (2) GPR128 (e.g., in frontal cortex at chr3:100,371,871; *P* = 8.32x10^-70^). Another interesting example in NABEC is a common CNV-QTL with MAF ~16% in the KANSL1 gene (e.g., chr17:44,169,808; *P* = 3.07x10^-63^). KANSL1 is a chromatin modifier gene known for histone acetylation, enhancer regulation and a causal gene for the 17q21.31 microdeletion syndrome^35^. Further details regarding the complete set of results containing CNV-dosage analysis for all brain regions across all cohorts is available in the data availability section.

### NMF analysis in UKBEC Omnichip dataset

**Fig. 5:**
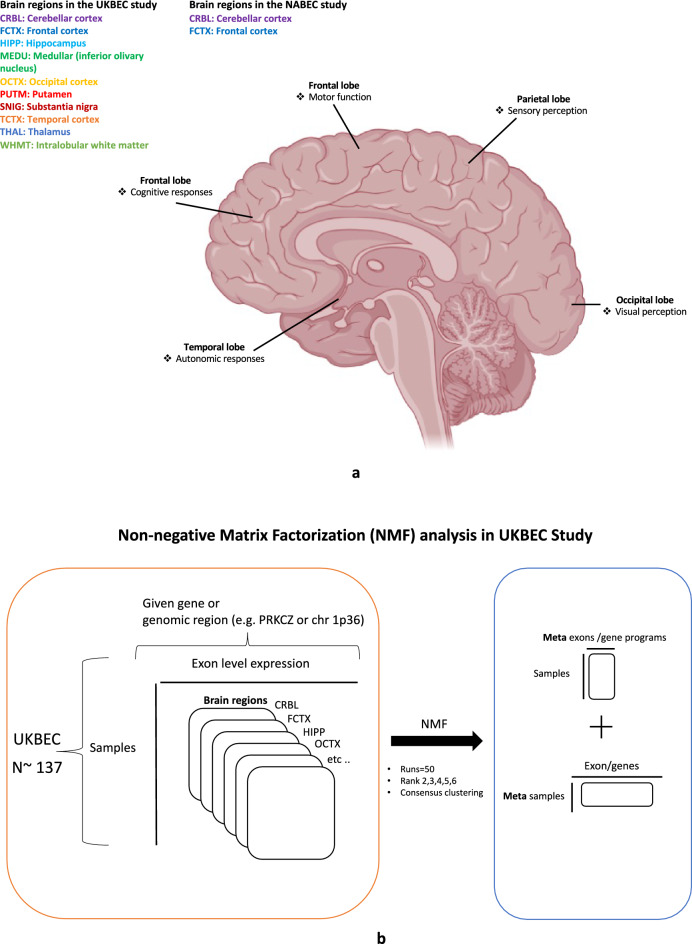
NMF analysis in the UKBEC study. (**a**) Summary of the brain regions analysed in the UKBEC and NABEC studies. (**b**) Overview of the NMF deconvolution analysis in the UKBEC gene expression dataset.

**Fig. 6:**
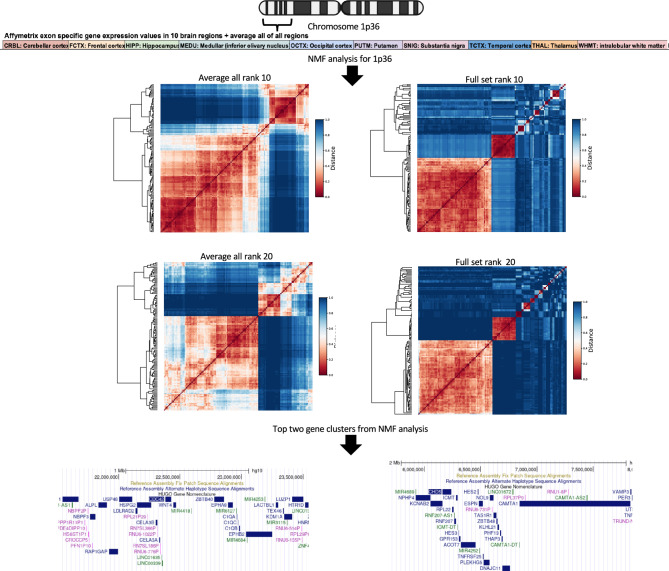
NMF gene clusters. Overview of the two gene clusters uncovered through the NMF analysis in the UKBEC gene expression dataset. The average-all expression matrix is derived by averaging gene expression data across the ten brain regions whereas the full-set expression matrix refers to a combined gene expression matrix for all brain regions.

Application of the non-negative factorization (NMF) method allowed us to deconvolute the UKBEC gene expression dataset from 10 regions into meta exons or genes (also referred to as hidden genes or patients in the literature). This transformation of gene expression data can be biologically interpreted as exons or genes which are consistently over or under expressed in different regions of the human brain (Fig. 5a, b). For the 1p36 region we ran the NMF algorithm on two expression matrices. One is derived by averaging the gene expression values across 10 regions (denoted as aveALL) and second is a combined expression matrix including all brain regions (denoted as full set). On analysing the relative frequency of individual exons in these results (NMF consensus clustering) we discovered two gene clusters in the chromosome 1p36 region (Fig. 6**; **Supplementary Table 9). The first cluster was located around CDC42 gene with nearby genes (~1 mega base pair window) such as RAP1GAP, USP48, HSPG2 and LUZP1. The second cluster was found around CHD5 and included nearby genes KCNAB2, CAMTA1 and ACOT7. Of note, similar to GNB1 both CDC42 and CHD5 were found to be highly methylated (methylated read count >700) in the BOCA-FR cancer cohort (Supplementary Table 5). Further, application of NMF algorithm for every gene individually (with 5-kilo base pairs window around gene boundary) allowed us to see which exons are consistently over or under expressed across various brain regions. In summary, first we applied the NMF deconvolution to the chromosome 1p36 region in the UKBEC Omnichip dataset with rank 10 and 20 for two expression matrices derived from all brain regions, and then separately to all known human genes (rank 2-6, for (a) 2 derived expression sets and for (b) ten brain regions separately; see methods; all NMF results are available in the data section). Additionally, in the UKBEC dataset we performed a secondary co-expression analysis using the weighted gene co-expression network algorithm (WGCNA)^36^ in the chromosome 1p36 and 9q22 regions. Here, the results indicated the presence of two gene clusters for both (Supplementary Figs. 16-17). In the chromosome 1p36 region one of the main clusters contained CHD5 and the second one had genes near GNB1 (Supplementary Table 10).

## Discussion

Our results complement the recent large-scale studies in epilepsy GWAS^1,37^ by filling the gap for small and intermediate CNVs and further elucidating its effect on phenotypes like seizures and drug-response. We found that population level analysis of CNVs leveraged more power to detect previous findings such as GNB1 for seizures and neurodevelopmental disorders^38^ and in addition successfully uncovered new genes and loci. For instance, our seizure-associated CNV locus in GNB1 located at chr1:1,745,726-1,810,090 in SANAD complements the earlier reported^38^ downstream germline and somatic single nucleotide variants (SNVs) near exon 6 of GNB1. Existing protein structures depicting the interaction of GNB1 with HTR1B and growth hormone-releasing hormone receptor (GHRHR) further strengthen our GNB1-CNV results in SANAD. These observations highlight the power of population-aware methods for CNV genotyping^39^. This is significant since unlike cnvHap the current methods for CNV detection from bead array chips usually apply hidden Markov models (HMMs) on a sample-by-sample basis. Due to this one may fail to capture the population-level information which could be leveraged for modelling the dosage landscape in the human genome^40^. Some highlights of our new genes for epilepsy phenotypes like seizure counts and seizure frequency included PRKCZ, HEATR1, TRDN, CNTNAP3, AEBP2 and GABRB3 (Supplementary Figure 18 shows GNB1-GABRB3 interactions).

PRKCZ is a calcium (Ca^2^^+^) dependent gene known for memory function or long-term potentiation^41,42^. HEATR1 (also known as BAP28) is required for pre-ribosomal RNA transcription by RNA polymerase I which is known to cause brain abnormalities in zebrafish^43^ and drosophila^44^. TRDN leads to muscle contraction by Ca^2+^ release and is a causal gene for Cardiac arrhythmia syndrome with or without skeletal muscle weakness (CARDAR)^45–48^ (https://www.malacards.org/). The relevance of CNTNAP3^49,50^ and GABRB3^51–53^ for neurological diseases is well documented in current literature. Lastly, the AEBP2 gene codes for a subunit in the core Polycomb repressive complex 2 (PRC2)^54,55^ which affects histone H3K27 (H3K27me3) trimethylation^56^ on the chromatin leading to long-term epigenetic silencing (also referred to as cellular memory). In our results, the role of epigenetics is further highlighted by the 10x levels of methylation seen in the seizure-associated locus in GNB1 for BOCA-FR or separately in the methylation fluctuations seen in HFM1 gene for paediatric brain cancer patients. Further, the distinct long-range methylation waves seen in the chromosome 1p36 region for multiple cancer cohorts strongly indicate that epigenetically driven co-regulation of genes (e.g., synergistic regulation) or transcriptional heterogeneity is likely to be a critical factor for maintaining normal brain functions and homeostasis. However, at present the epigenetic axis of gene regulation through enhancer or promoter interactions^57–59^, topologically associating domains (TADs)^60,61^ or the newly observed methylation waves in our study, could affect the phenomenon of co-localisations of GWAS or eQTL association signals (e.g. the chromosome 1p36 region or the UKBEC chromosome 9q22 gene cluster), remain to be further elucidated. Of note, our methylation results also reflect some of the original findings from the encyclopedia of DNA elements (ENCODE) project^59^ where the authors reported differential methylation to be prevalent in many different cell types and additionally indicated its preference for chromatin accessibility. Lastly, based on our observations in SANAD, we note that variation in some genes like PRKCZ or TRDN may lead to susceptibility of secondary symptoms (in epilepsy or other neurodevelopmental disorders) e.g., memory loss or arrhythmia.

Our CNV results for UKBEC and NABEC cohorts complement the current SNP-QTL results^9,62,63^ in current literature. To the best of our knowledge, our study is one of few which has elucidated the dosage effect of small CNVs in different regions of the brain in a comprehensive manner. Further, our replication model based on LRR signal was consistent in uncovering meaningful genes for neurology and important brain functions. For instance, in the case of the SCN1B gene, which is a known candidate gene for LGS, we did not detect any CNVs in all our cohorts (which could be due to low probe density). However, by using the LRR based model in the UKBEC dataset we uncovered suggestive signals in particular brain regions such as putamen, white matter, and occipital cortex (Supplementary Table 11).

The convergence of top hits of CNV-QTL in UKBEC Omnichip and Immunochip data to the chromosome 9 gene cluster which also happens to harbour the GABBR2 gene (G protein-coupled receptor 3 family and GABA-B receptor subfamily) strengthens the clustering phenomenon earlier reported in the original UKBEC SNP-eQTL study^9^. Further experiments aimed at better understanding the causal mechanisms behind such clustering could potentially provide new information related to human brain function and warrants further investigations. Importantly, based on our observations we suggest that in-tandem transcriptional regulation of co-located genes in the genome could be an important mechanism by which important molecular functions are carried out in the brain. To this end, our NMF results from the UKBEC expression dataset hint that transcriptional sense and relative position of exons (i.e., exons which are transcribed early along the RNA polymerase machinery) might have higher or lower overall RNA concentration in the cell (Supplementary Table 12). This differential expression of exons which could potentially affect splicing might be interpreted as RNA amplitude for a given exon in a tissue. The methylation waves seen in genes like HFM1, WWOX etc and clustering of CNV-phenotype signals in SANAD e.g., GNB1 region on chromosome 1p36 or the chromosome 9 CNV-QTL cluster in UKBEC results, provides suggestive evidence for the existence of such mechanisms. However, this remains to be further elucidated and experimentally validated. Lastly, the reciprocal CNV-dosage effect we observed in the HTR3B and DRD2 genes and its link to the CRHR2-INMT metabolic pathway is potentially a new genetic finding. Further elucidation of this molecular axis at a population level and possibly through epigenetic profiling, could potentially shed light on how the brain maintains the homeostasis of neurotransmitters and hormones through genomic regulation of serotonin, dopamine and cortisol. Based on these findings and the results for GNB1 we conclude that CNVs through its effect on protein receptor complexes has an important role to play in neurological diseases and maintaining homeostasis in the brain.

### Limitations of this study

Leveraging our primary findings in GNB1 for SANAD, we would like to highlight that our results here have several important caveats. First, though we have reported strong evidence of CNV-seizure activity in GNB1, these results are not yet applicable for clinical translation in epilepsy patients. We suggest that the ideal way forward should be based on, first, next-generation deep sequencing or PCR based validation for CNVs in GNB1 and the chromosome 1p36 region, and next, further multi-omics profiling. This is especially relevant for profiling CNVs since our results indicate that high epigenetic activity like methylation is likely to affect the dosage effect of CNVs on gene expression and hence also on the phenotype. We would also like to highlight that many of the additional regulatory features which were explored in the original ENCODE study^59^ (e.g. chromosome conformation capture (3C)-based techniques for probing long-range interactions or DNA-protein interactions (e.g. ChIP-seq)) is currently lacking from our analyses presented here. In addition, our results for the meta-analysis of epilepsy cohorts currently lean towards SANAD, hence requiring additional replication efforts with sufficient statistical power in the future. The suggested use case for our current candidate gene lists is generating new hypotheses for understanding the molecular mechanisms of brain function and phenotypes e.g., shedding light on the molecular basis of seizure frequency. Based on the UKBEC study results, one may also consider designing new experiments for better understanding the role of protein receptor complexes in the neurology e.g., experiments to further elucidate the phenotypic effects of reciprocal dosage effect of DRD2 and HTR3B.

## Methods

### Study cohorts

#### Epilepsy cohorts

Similar to our previous study^4^, in our main CNV analysis in epilepsy we adopted a prospective cohort design instead of case-control designs. Our discovery cohort consisted of 916 subjects from the Standard and New AED (SANAD) clinical trial^8^ and a secondary replication cohort consisted of 380 subjects recruited from the Royal Melbourne and Austin hospitals in Australia. A brief description of all clinical variables analysed in all cohorts, including common phenotypes for meta-analysis is described in the Supplementary Table 13. The main phenotype categories analysed can be broadly divided into seizure related phenotypes (e.g., seizure frequency, total number of seizures etc) and 12-month remission to AED medication (responders vs non-responders). Epilepsy and seizure classification were based on the latest ILAE (The International League Against Epilepsy) guidance. The main types of epilepsy in our study consisted of focal, generalized and unclassified epilepsy. Genotyping of SANAD and Australian samples was carried out using Illumina 660 bead chips at the Sanger Centre at different points of time. Further quality control measures based on heterozygosity, sample call rates and relatedness were carried out on the raw intensity data. Additional information on genotyping and quality control for these cohorts can be found in our earlier reports^4^.

### Cancer studies

The International Cancer Genome Consortium (ICGC) was a collaborative effort which collected, harmonized and made multi-omics cancer data (including TCGA and Sanger Cancer genome project) accessible to the public through its data portal^64^ (retired in June 2024). We previously downloaded copy-number variation calls, whole-genome bisulfite sequencing for methylation and RNA-seq data for the following projects: Soft Tissue cancer- Ewing sarcoma France (BOCA-FR) (n=107), Chronic Lymphocytic Leukemia Spain (CLLE-ES) (n=551), Malignant Lymphoma Germany (MALY-DE) (n=252), Pediatric Brain Cancer Germany (PBCA-DE) (n=541) and Pediatric Brain Tumor Multiple subtypes USA (PBCA-US) (n=290). Here, we have analysed and presented results from these five cohorts.

### The UKBEC and NABEC study

The UKBEC study consists of 134 individuals of European descent who were confirmed to be neuropathologically normal during life and had a median age of 59^9^. 74.5% of patients are male and the most common cause of death in these individuals was heart attack. Further details about tissue collection and genotyping have been reported in detail in earlier studies^9^. Briefly, the whole transcriptome was available for 10 brain regions based on their relevance to human disease and reported to exhibit high expression profiles. RNA was extracted from post mortem brain tissues with randomization and checked for quality. Next, processed RNA was analysed through the Affymetrix Exon 1.0 ST array. Next, all arrays were processed by robust multi-array average normalisation and log2 transformed in two different ways. Genomic DNA from samples from the post-mortem brain tissue was processed using Qiagen’s DNeasy kit and subsequently genotyped on (1) Illumina Omni-Quad bead chip and (2) a custom Immunochip designed to fine map autoimmune disorders. GenomeStudio v.1.8.x was used for processing intensity data from which log*R* ratio (LRR) and B-allele frequency (BAF) was derived and exported for CNV analysis. The NABEC study^10,11^ consists of approximately 360 individuals of European descent and free from any neuropathological disorders. RNA was quality checked and extracted for hybridization onto Human HT12v3 expression bead chips. Raw gene expression data was further transformed using cubic spline and log transformed. Next, expression values were re-mapped using ReMOAT onto human genome build 19 and annotated with genes with reliable data and free from common polymorphisms. Genomic DNA for the NABEC study was extracted and genotyped on Illumina Infinium HumanHap550 chips. Like the UKBEC study, intensity values LRR and BAF were processed using genome studio software and exported for subsequent CNV analysis.

### CNV detection and genotyping through cnvHap

Similar to our earlier studies^39,40^ we relied on the cnvHap algorithm^29^ as the main CNV discovery method. cnvHap is a multi-platform CNV-SNP haplotype based CNV detection algorithm which uses fluorescence signal intensity (referred to as Log-R ratio or LRR) and relative signal intensity between the two SNP alleles (referred to as BAF or B-allele frequency) to simultaneously discover and genotype CNVs. cnvHap was shown to have more sensitivity in detecting smaller common CNVs with high genotyping accuracy. Due to its population-aware mode of model training it was pragmatic to use this method for CNV-phenotype association analyses in large cohorts which were originally genotyped on bead array chips for SNP-GWASs or SNP-eQTL studies. All cohorts analysed in our study including for epilepsy (SANAD and Australia), UKBEC and NABEC cohorts were genotyped on different versions of Illumina bead array chips, hence processed through a common CNV pre-processing pipeline. Briefly, for each cohort, the two intensity measurements, LRR and BAF were exported from Illumina genome studio software as final reports. Next, the exported intensity measurements were normalised for GC content and further regressed out from LRR values. Genomic wave effects were removed by fitting a localised loess function. In the main cnvHap analysis, joint CNV-SNP haplotype structure information was incorporated to refine CNV predictions. Further, based on allele frequencies, expected CNV genotypes were calculated for subsequent MultiPhen based association analyses. First, we fine-tuned our CNV analysis pipeline to reproduce known common CNVs in our cohorts. We chose the WWOX intronic deletions as reported by the gnomAD database in the region chr16:78,371,638-78,385,000 (GRCh37/hg19) which has a deletion and a multi-CNV with allele frequency of 34% to 54% respectively (Supplementary Figs. 19-20). Missense mutations in exons of WWOX have been reported to be associated with highly pathogenic WOREE syndrome in epilepsy, which usually occurs in young children and has a very poor prognosis. In SANAD we re-discovered this intronic CNV in WWOX as common deletions with an allele frequency of 47% spanning the region chr16: 78,373,644 - 78,384,121. Manual inspection of cluster plots of LRR and BAF showed distinct heterozygous deletions spanning more than 40 contiguous probes, which was highest amongst all CNVs detected in our cohorts. This contiguity of probes was also reflected through significant association results for epilepsy drug-response phenotype in the MultiPhen LRR based analyses of SANAD (without cnvHap calls). Of note, our replication cohort from Australia had batch effects and around 30% of the samples were excluded due to quality control issues in our CNV analyses. Unlike SANAD, the resulting sample size from Australia (n~280) thus had limited statistical power to detect and genotype common CNVs on a broad allele frequency spectrum at genome wide significance level. We calculated that based on a population-level prevalence of epilepsy at 1% and additive genetic model, we would roughly require a sample size > 1,000 to achieve 80% power at standard GWAS significance threshold. However, data from the Australian cohort independently and in the meta-analysis with SANAD was able to verify and replicate many of our primary findings. In addition, they were enriched for genes related to neurological conditions, hence reported in our analysis. For the meta-analysis of SANAD and Australian cohorts, we utilised the multi-platform integration feature of cnvHap, where intensity values for genotyping probes in both our cohorts were modelled jointly by the HMM model for predicting CNV genotypes.

### cnvHap parameters and quality control

All parameters used for CNV calling in cnvHap are listed in Supplementary Table 14. Briefly, for CNV discovery in SANAD the important parameters included *r_mean* value of -4.5;-0.3;0;0.305;0.528;0.702;0.8434;0.9848;1.126 (which controls the mean LRR-BAF cluster positions in the HMM model), intensity variance threshold of 0.25 which were further corrected for GC and loess, 15 iterations of the expectation-maximisation (EM) step and HMM emission group set to Illumina^29^. We used the same parameters for the Australian cohort and in the multi-platform (joint analysis) of SANAD and Australian cohorts. In the latter analysis the equalise group (reference set of probes for the HMM model) flag set to SANAD. Further details regarding the moving genomic window (chromosome wise genomic coordinates) used for the underlying cnvHap HMM model is listed in Supplementary Table 14**b**. Next, after CNV calls were generated by the HMM model, expected CNV genotypes were calculated for every genotyping probe and subsequently used for all CNV-phenotype association analyses (performed through the MultiPhen method). Briefly, if at a particular probe a sample has CNV genotype assigned as 1 (heterozygous deletion) with probability of 0.8 then the expected CNV genotype is calculated as: 1*0.8+2*0.2=1.2. A final CNV genotype, referred to as “countAll” in the cnvHap output was calculated and used in all our association models. The countAll value ranges from 0 to 4 and is obtained by summing over the expected CNV genotype values for deletions, copy-neutral and duplication genotypes. Of note, in addition to the *countAll*, cnvHap also produces two other values of expected CNV genotypes 1) *state.0 or* deletion-only model and 2) *state.2 or* duplication-only model. Here, we have only reported results for the countAll values. Further, to disentangle the effect of deletions and duplications at the same locus one can stratify the countAll CNV genotypes into deletions (e.g. samples with countAll values < 1.7), copy-normal and duplications (countAll > 2.5). Next, based on this sample stratification one can further apply non-parametric statistical tests to compute P values for phenotypic distributions. Further, all results reported by cnvHap had at-least average certainty (posterior probability) of 0.5 and the total number of samples included in the final association analyses after quality control was (as it varies probe-by-probe) approximately ~ 620 for SANAD, Australia ~ 290 and ~ 887 for the meta-analysis (see data section for all association results). All genes of biological interest discussed here had MAF of 1% or more.

### Association models

One of the main goals of SNP-eQTL studies is enabling better understanding of GWAS loci. Here, in our study design we aimed at emulating this approach by finding new CNV loci for epilepsy phenotypes in SANAD and Australian cohorts and then leveraging CNV-eQTL analysis in normal human brain regions from the UKBEC and NABEC studies for understanding CNV signals for epilepsy. The CNV-eQTL results and gene NMF programs (described later) from the UKBEC and NABEC cohorts can be used as an independent resource for replication and validation of a priori disease hypothesis as well for other neurological diseases. Our association analysis for epilepsy cohorts, UKBEC and NABEC consisted of six different linear modes implemented in the MultiPhen software^13^. These models were based on two approaches of CNVs detection (1) Expected CNV genotypes derived from cvnHap and (2) Log-R ratio based raw intensity measurements (i.e., without using cnvHap and for secondary validation of CNV-phenotype signals). Next, two modes of CNV signals were analysed using 3 linear models: (a) standard univariate model (phenotype ~ CNV genotypes /LRR) (b) MultiPhen joint model ( CNV genotypes/LRR ~ Phenotypes) and (c) MultiPhen joint model with backward variable selection (CNV genotypes/LRR ~ Phenotypes (subset). Of note, in our analysis only the results of CNV association with epilepsy phenotypes in SANAD is the main discovery results and all other reported results from Australia, UKBEC and NABEC are meant to be used as replication results with a priori hypothesis. We used 5 LRR principal components and gender as covariates for the SANAD cohort and only gender for the Australian, UKBEC and NABEC studies. The main criteria for choosing parameters for cnvHap and covariates for MultiPhen were reproducing allele frequency of common CNVs e.g., matching WWOX deletion frequency from gnomAD CNV results and reproducibility of known genes for neurological diseases. Lastly, like our previous study^40^, the following approach for multiple-testing correction was applied for all P values reported here. Briefly, we used a modified Šidák method for calculating the net effective number of tests (M) based on the degree of correlation structure in the phenotype matrix.

$$_{{{\text{M effective }} = { 1 } + \, \left( {{\mathrm{M}} - {1}} \right)({1} - {\text{Var }}\left( {\lambda {\text{ observed}}} \right) \, /{\text{ M}})}}$$

Here, λ is the eigen decomposition of the correlation matrix (see original publication^13^ for more details).

### Pseudocodes for MultiPhen models

1) Standard univariate model for GWAS

$${\text{glm }}\left( {{\text{phenotype vector }}\sim {\text{covariates }} + {\text{ variables}},{\text{ family }} = {\text{ family}},{\text{ weights }} = {\text{ weights}},{\text{ offset }} = {\text{ offset}}} \right)$$

2) Multiphen reverse regression or joint model

$${\text{glm }}\left( {{\text{variables }}\sim {\text{ phenotype vector }} + {\text{ covariates}},{\text{ family}} = {\mathrm{family}},{\text{ weights}} = {\mathrm{weights}}} \right)$$

3) Multiphen with variable selection or joint model with variable selection
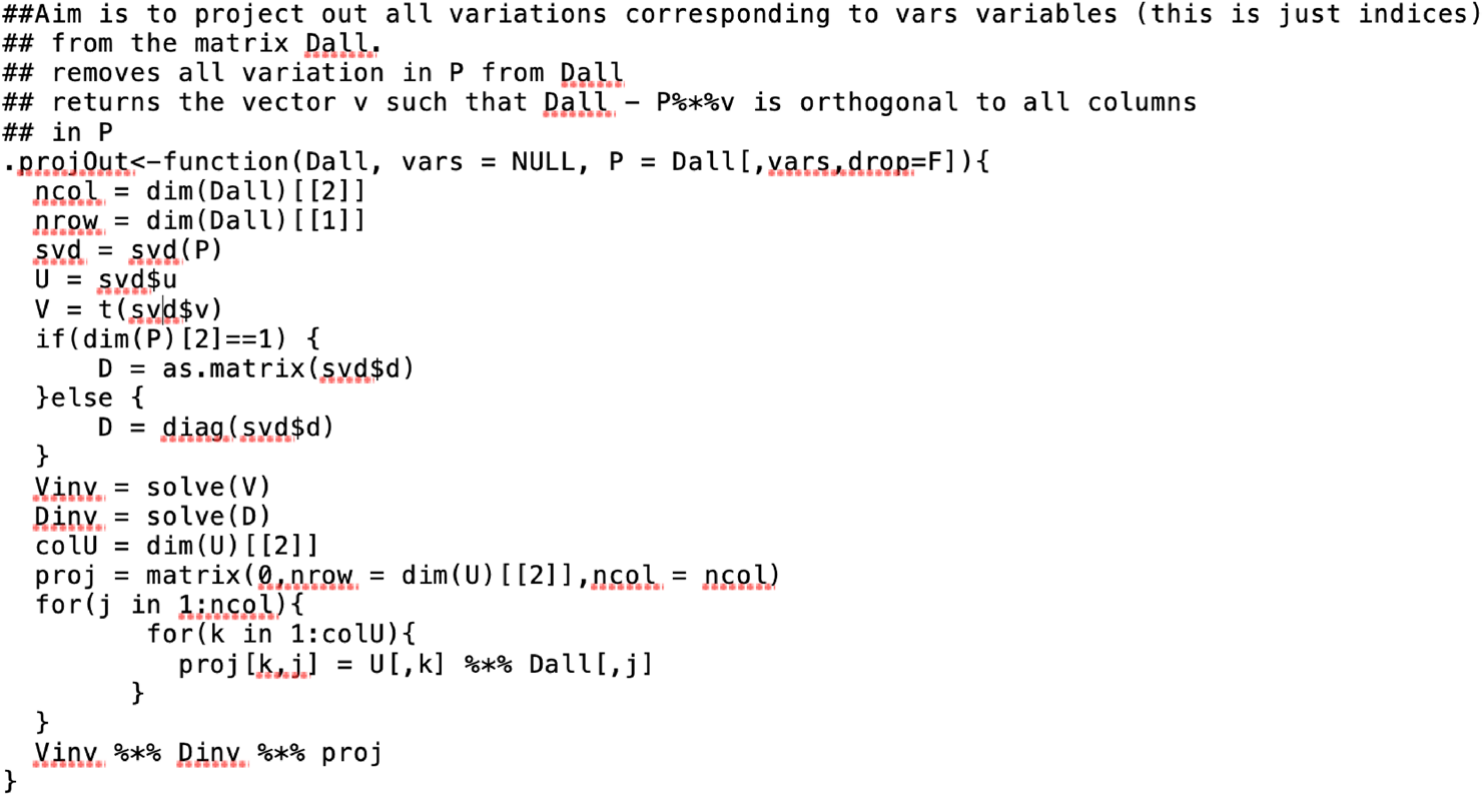


### NMF gene programs

Non-negative matrix factorization is a popular method in data science for deconvoluting complex data including images and gene expression data. It has been successfully used for finding subtypes of cancer and more recently for finding common gene programs across multiple single cell gene expression data. Here, we have measurements for all human genes and their corresponding exons in ten brain tissues with an additional set derived from the average expression across all ten brain tissues. With the aim of finding common exons or genes which are consistently over expressed or under expressed in these 11 sets of gene expression data matrices, we leveraged the NMF algorithm to find meta exons/genes and corresponding meta patients. We performed this analysis using two approaches. First, a gene-by-gene approach where for all 20, 000 human genes we extracted probes within a 5-kbps window around each gene and derived 11 sets of brain tissue expression data. Next, for these matrices we ran the NMF algorithm 50 times for ranks 2-6. Next, from the output of multiple NMF runs we extracted meta exons or genes using consensus clustering methods as described here^65^. Next, from these results we counted how many times each probe occurs in each gene program for ranks 2-6. Here, frequency of these counts may be interpreted as relative importance of exons which might have deeper biological or disease relevance. In the second approach instead of a gene boundary we ran the NMF analysis over a genomic region e.g. chromosome 1p36 region.

## Supplementary Information

Supplementary Information 1.

Supplementary Information 2.

Supplementary Information 3.

Supplementary Information 4.

Supplementary Information 5.

Supplementary Information 6.

Supplementary Information 7.

Supplementary Information 8.

Supplementary Information 9.

Supplementary Information 10.

Supplementary Information 11.

Supplementary Information 12.

Supplementary Information 13.

Supplementary Information 14.

Supplementary Information 15.

## Data Availability

UKBEC study data is available on Gene Expression Omnibus as described by the authors of the original study. Microarray CEL files and processed data is under the accession number GSE46706. SNP data for UKBEC is available through dbGAP data access committee (DAG) after appropriate applications and approvals. All association results are available on Zenedo with the url: https://zenodo.org/records/15992704 Summary of results and data on Zenedo are as follows: 1.CNV and LRR results for SANAD 2.CNV and LRR results for Australian cohort 3.CNV and LRR results for SANAD and Australian cohort meta-analysis. 4.CNV and LRR results for UKBEC Omnichip - gene wise 5.CNV and LRR results for UKBEC Omnichip - split file/moving window 6.UKBEC Omni chip CNV/LRR QTL (gene-wise) results and heatmaps for GNB1 and HTR1B 7.UKBEC Omni chip CNV/LRR QTL (gene-wise) results and heatmaps for the chromosome 9q22 region 8.NMF analyses for all human genes in the UKBEC Omnichip 9.CNV and LRR results for UKBEC Immunochip data. - split file/moving window 10.CNV and LRR results for the NABEC cohort - split file/moving window 11.cnvHap breakpoints for all cohorts 12.An example input dataset for cnvHap with LRR and BAF values. (See github details for code)

## Materials availability

Samples and further materials related to the SANAD study, and the Australian Epilepsy cohort can be requested from the lead author or Dr Michael R Johnson, Department of Brain Sciences, Imperial College London, UK. Materials requests for the UKBEC and NABEC study should be addressed to the respective authors of the original study. UKBEC and NABEC data usage adheres to the original data usage and sharing guidelines. Prior ethical approval and written consent were obtained from all study subjects involved in the respective studies including SANAD, Australian cohort, UKBEC and NABEC studies.

## Code availability

Our CNV detection pipeline was run on a high-performance computing cluster running PBS job scheduler. Scripts used for CNV analysis through cnvHap and MultiPhen (version 2.1.8 ) are available on github: https://github.com/DE-Tisham/CNV-dosage. A custom executable java file (jar file) of cnvHap (used in our analyses) with demo input data is available on google drive: https://drive.google.com/drive/folders/1Gm04HFy07l7uB6DqQP8ojcnCP2fFzkYf?usp=drive_link. Source code for MultiPhen version 2.1.8 with example scripts and outputs is available at https://github.com/DE-Tisham/MultiPhenv2.1.8. All codes used to process and analyse data have been published earlier. The original source code is available at: Multiphen: https://github.com/lachlancoin/MultiPhen, cnvHap: https://www.imperial.ac.uk/people/l.coin, Figure 1c and Fig. 3b were generated through the Mol^*^ software^66^ available at https://www.npmjs.com/package/@3dbionotes/pdbe-molstar. This study did not generate new reagents or software code.
