# Supplementary material for: Dosage effect of copy number variation in epilepsy and ten regions of the human brain
Source: Sci Rep. 2025 Dec 4;15:45726. doi: 10.1038/s41598-025-28338-2 (PMC12753814; doi:10.1038/s41598-025-28338-2)
Supplement: Supplementary file 15 — Supplementary Information 15. [file 41598_2025_28338_MOESM15_ESM.pdf]

Supplementary material

Dosage effect of copy number variation in epilepsy and ten regions of the human brain

Tisham De[1,2,3], Lachlan Coin[3,4], Michael R Johnson[5]

1. **Department of Epidemiology and Biostatistics, School of Public Health, Imperial College, London, UK**
2. **Department of Genomics of Common Diseases, Imperial College London, UK**
3. **Department of Infectious Disease, Imperial College London, UK**
4. **Department of Microbiology and Immunology, University of Melbourne at The Peter Doherty, Institute for Infection and Immunity, Melbourne, Australia**
5. **Department of Brain Sciences, Imperial College London, UK**

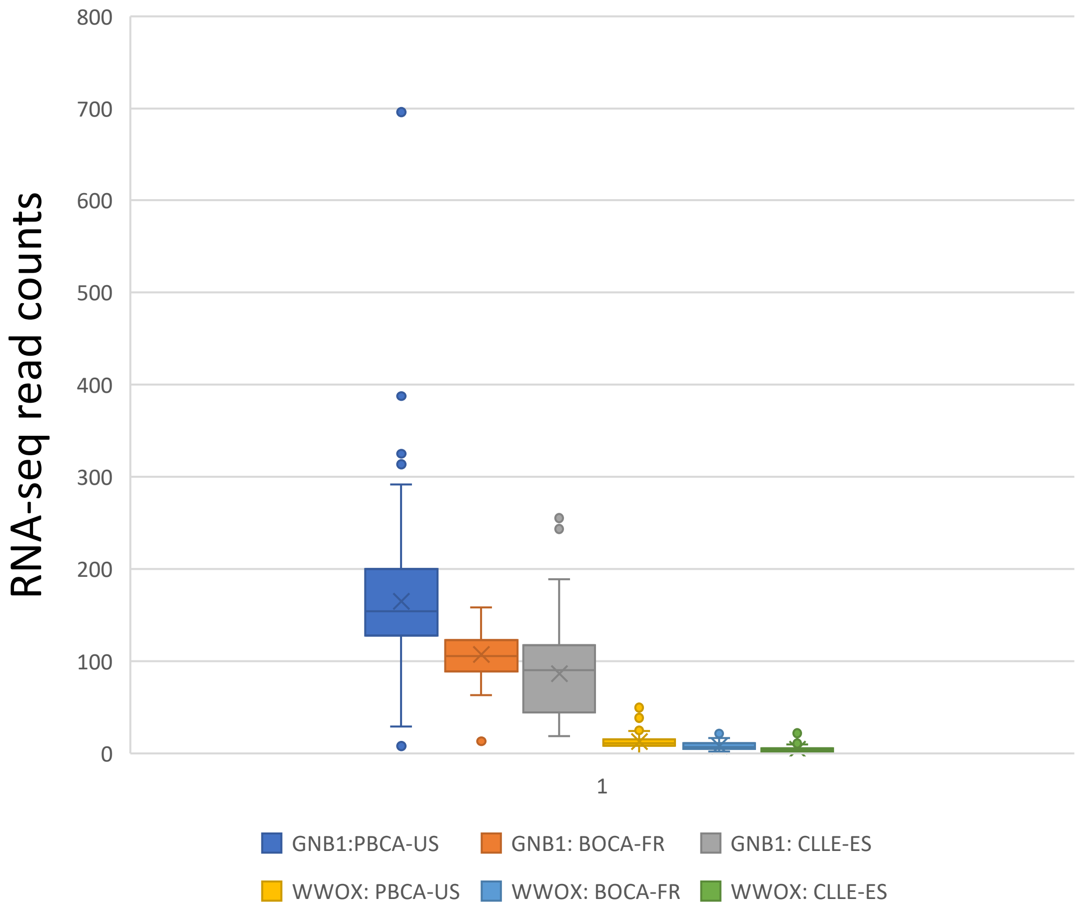

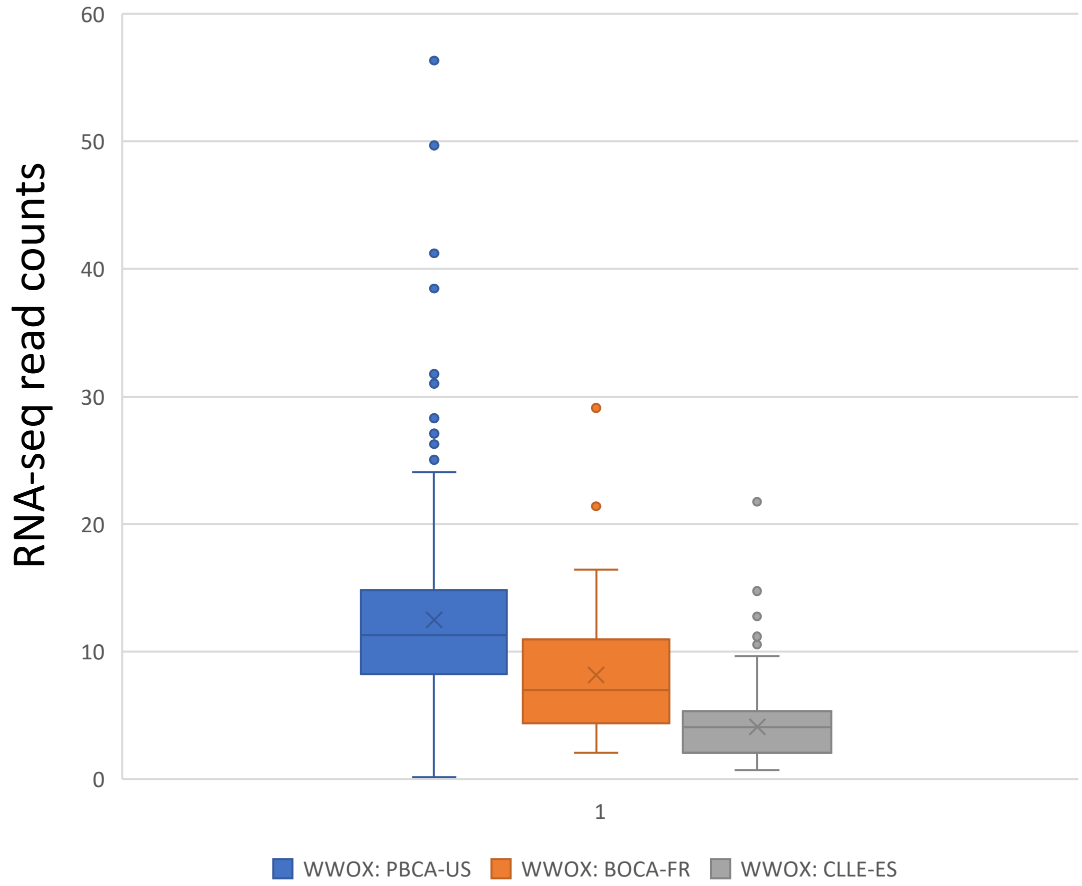

Supplementary figure 1a. RNA-seq read counts for GNB1. Figure showing raw RNA-seq read counts for GNB1 and WWOX in different cancer cohorts.

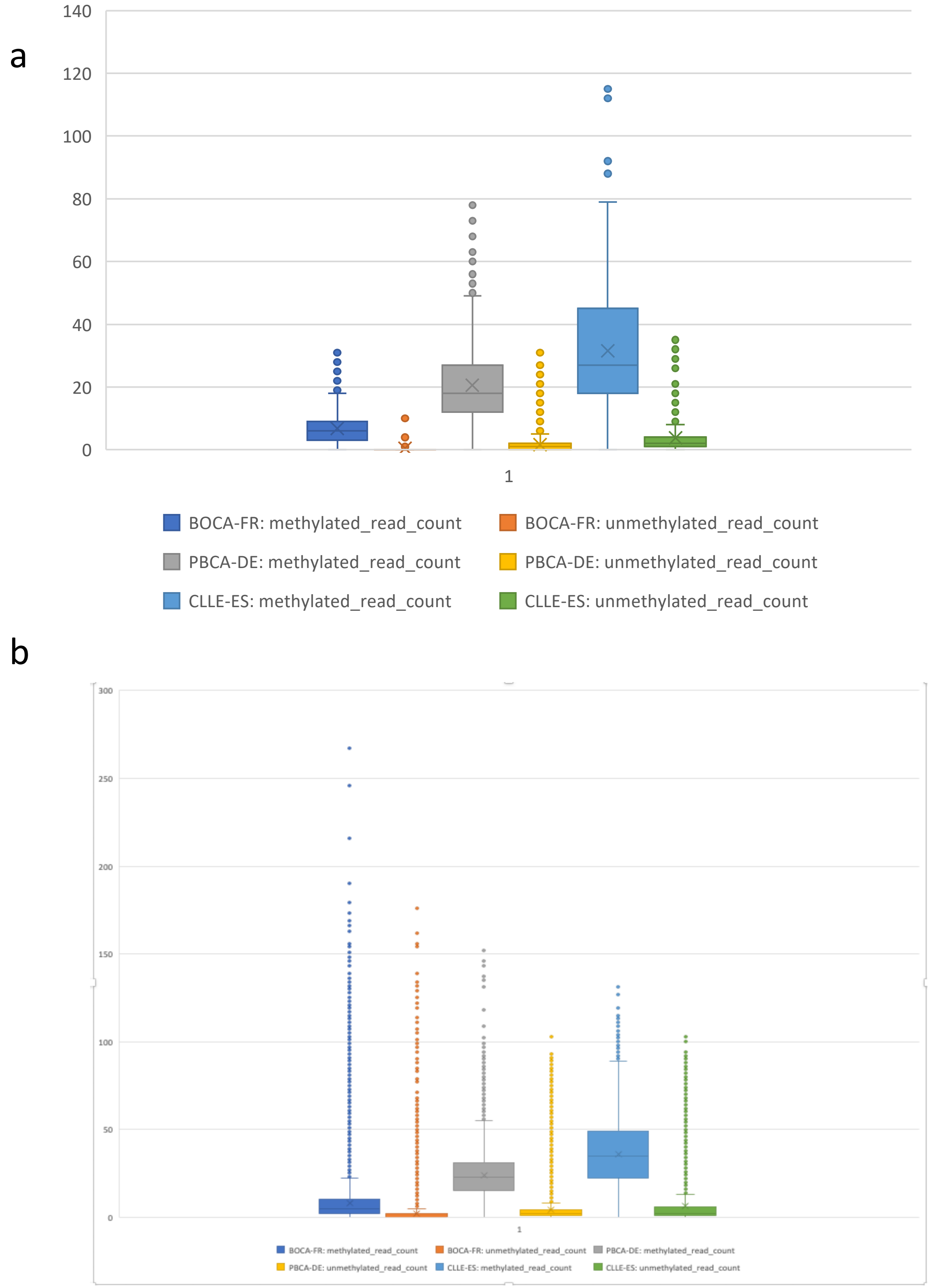

Supplementary figure 1b. Methylation data for WWOX. Figure demonstrating raw methylation read counts for the WWOX gene in different cohorts. (a) Methylation data for the common intronic deletion at chr16:78,371,638-78,385,000 (GRCh37/hg19). (b) Methylation data for WWOX excluding the intronic deletion region.

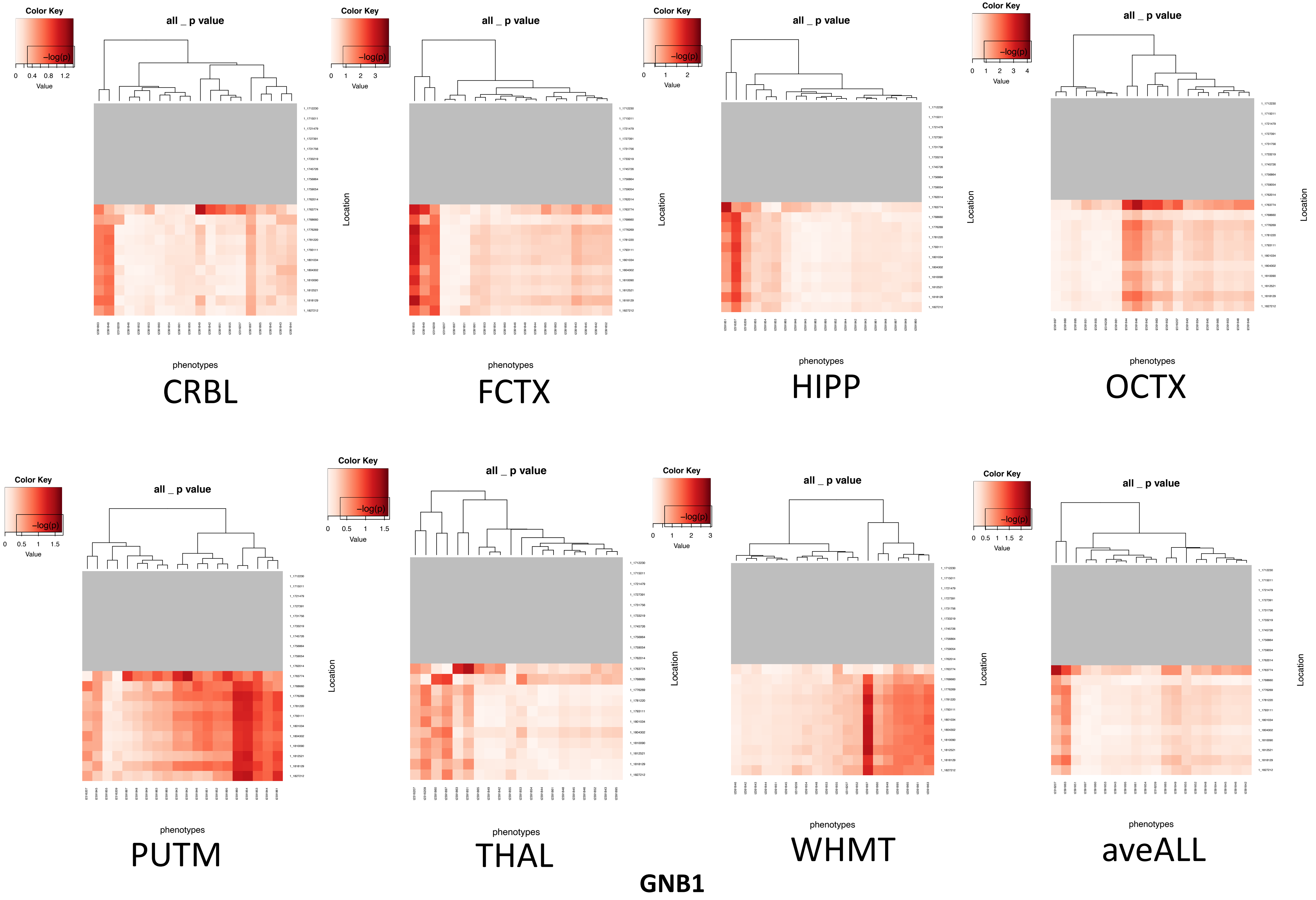

Supplementary figure 2. CNV-QTL in GNB1. Figure showing association results for CNV genotypes with exon level gene expression in GNB1 for different regions of the brain in the UKBEC omni dataset. The MultiPhen method used was standard univariate model. Grey colour denotes missing data or NA. This analysis was done on a gene-by-gene basis (see methods).

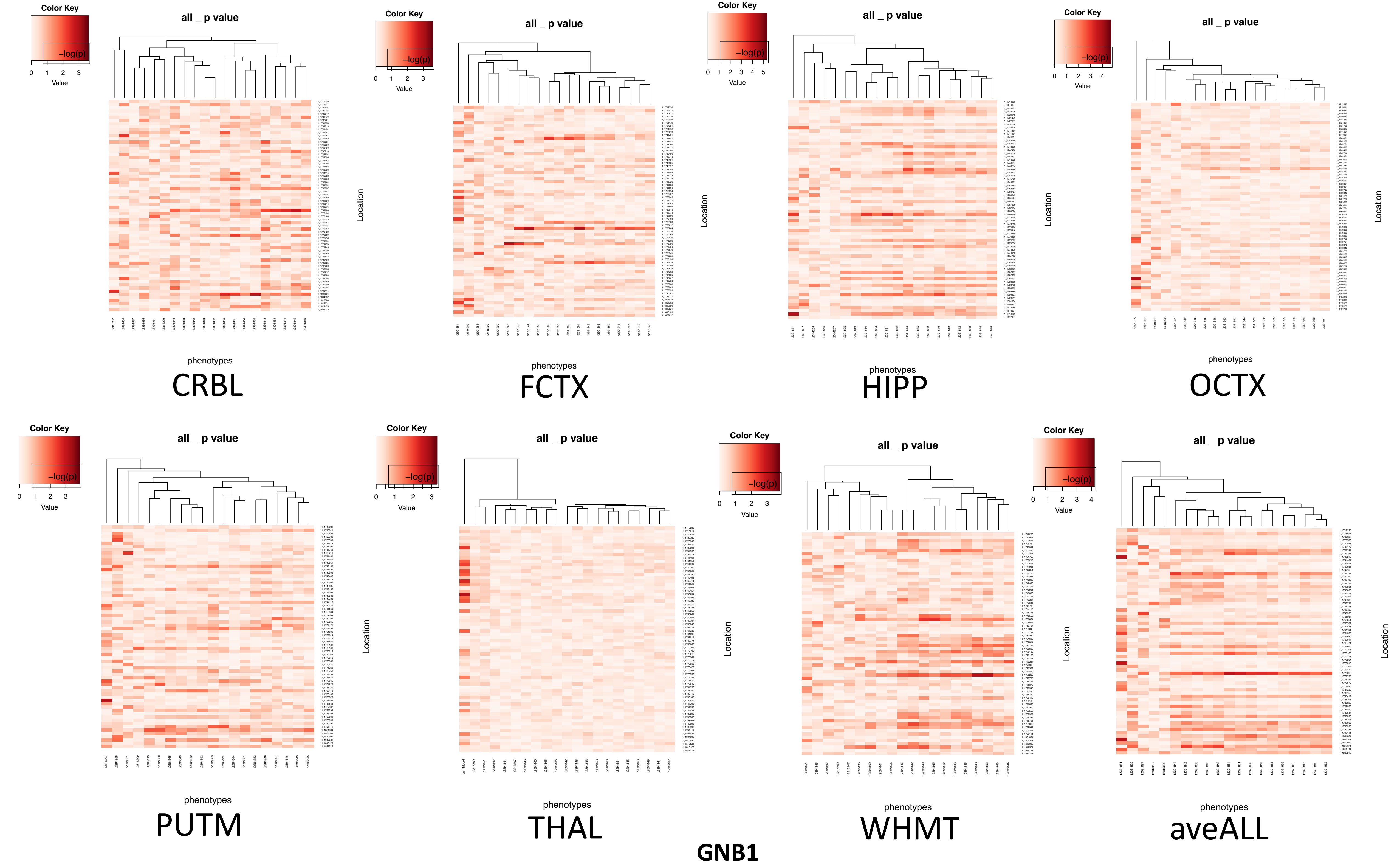

Supplementary figure 3. LRR-QTL in GNB1. Figure showing association results for log-R ratio with exon level gene expression in GNB1 for different regions of the brain in the UKBEC omni dataset. The MultiPhen method used was standard univariate model. Grey colour denotes missing data or NA. This analysis was done on a gene-by-gene basis (see methods).

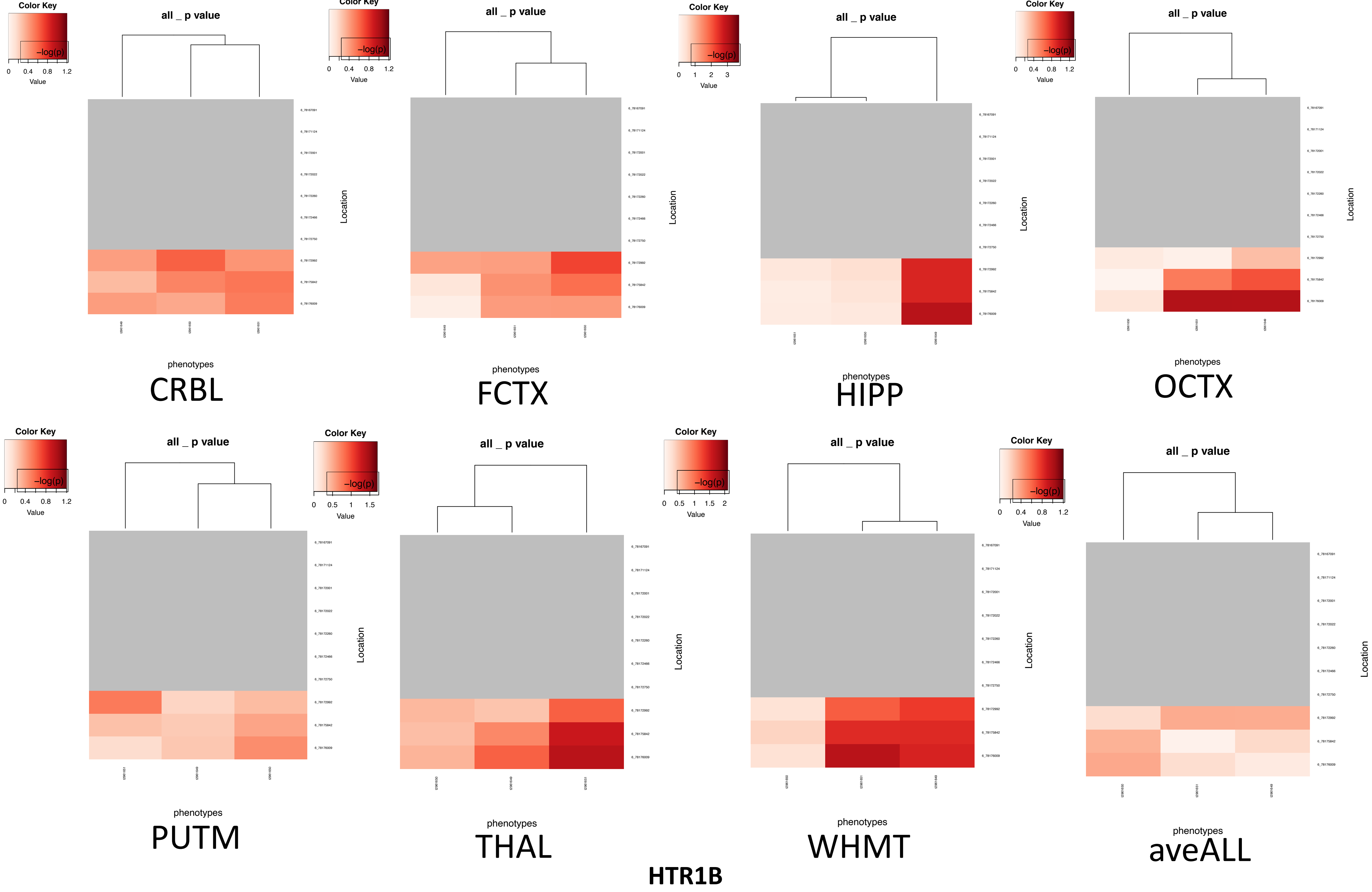

Supplementary figure 4. CNV-QTL in HTR1B. Figure showing association results for CNV genotypes with exon level gene expression in HTR1B for different regions of the brain in the UKBEC omni dataset. The MultiPhen method used was standard univariate model. Grey colour denotes missing data or NA. This analysis was done on a gene-by-gene basis (see methods).

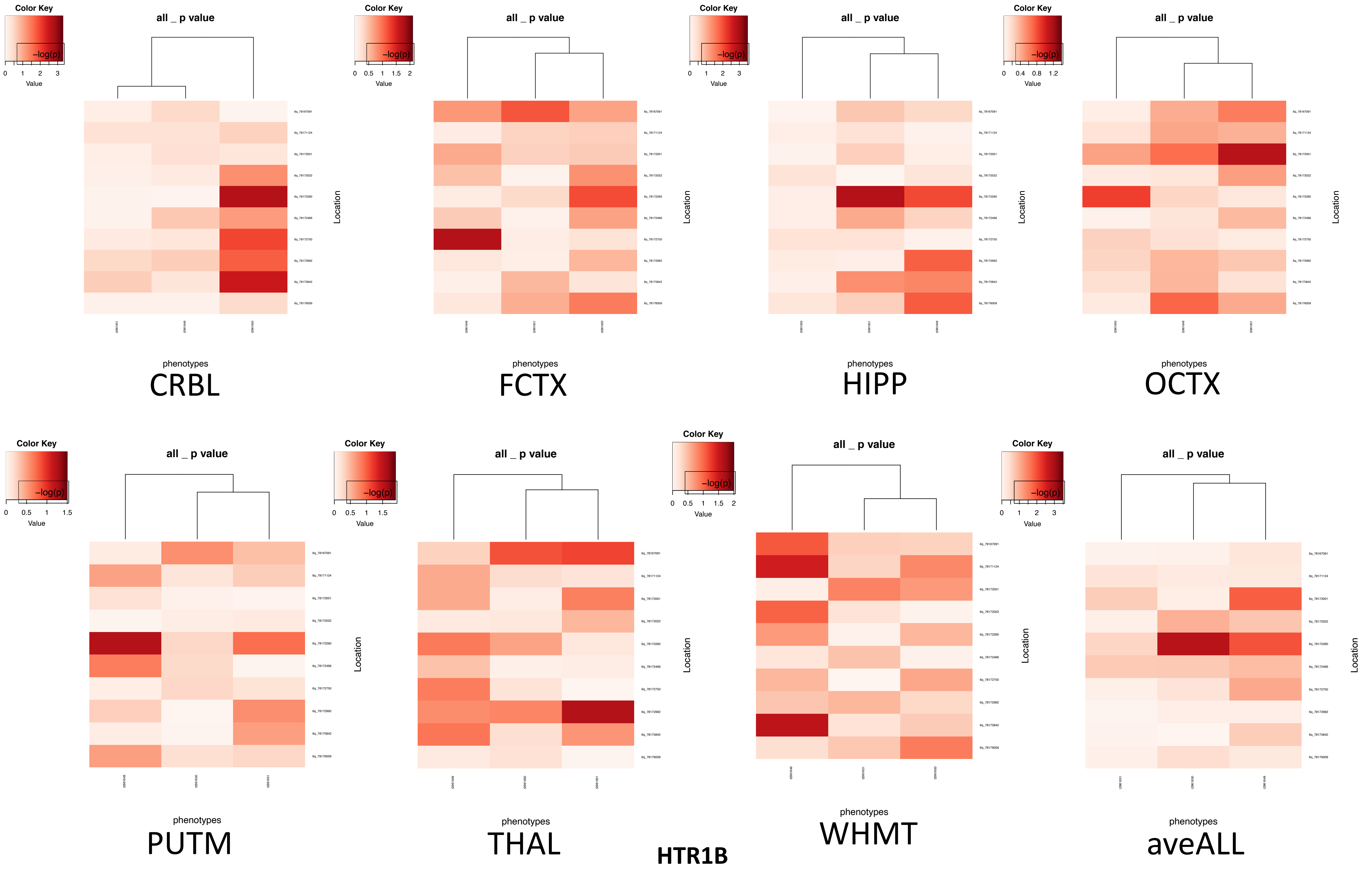

Supplementary figure 5. LRR-QTL in HTR1B. Figure showing association results for log-R ratio with exon level gene expression in HTR1B for different regions of the brain in the UKBEC omni dataset. The MultiPhen method used was standard univariate model. This analysis was done on a gene-by-gene basis (see methods).

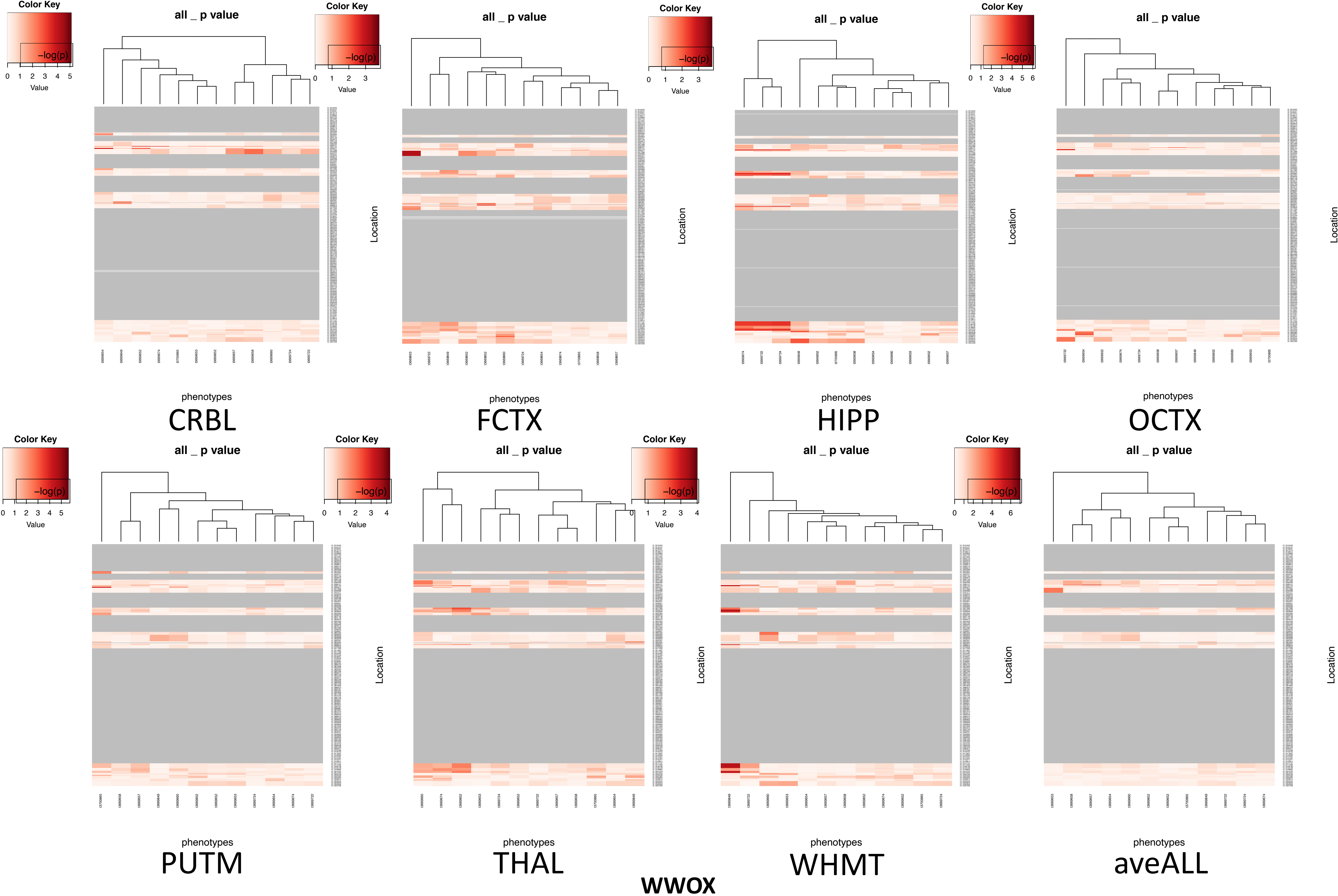

WWOX

Supplementary figure 6. CNV-QTL in WWOX. Figure showing association results for CNV genotypes with exon level gene expression in WWOX for different regions of the brain in the UKBEC omni dataset. The MultiPhen method used was standard univariate model. Grey colour denotes missing data or NA. This analysis was done on a gene-by-gene basis (see methods).

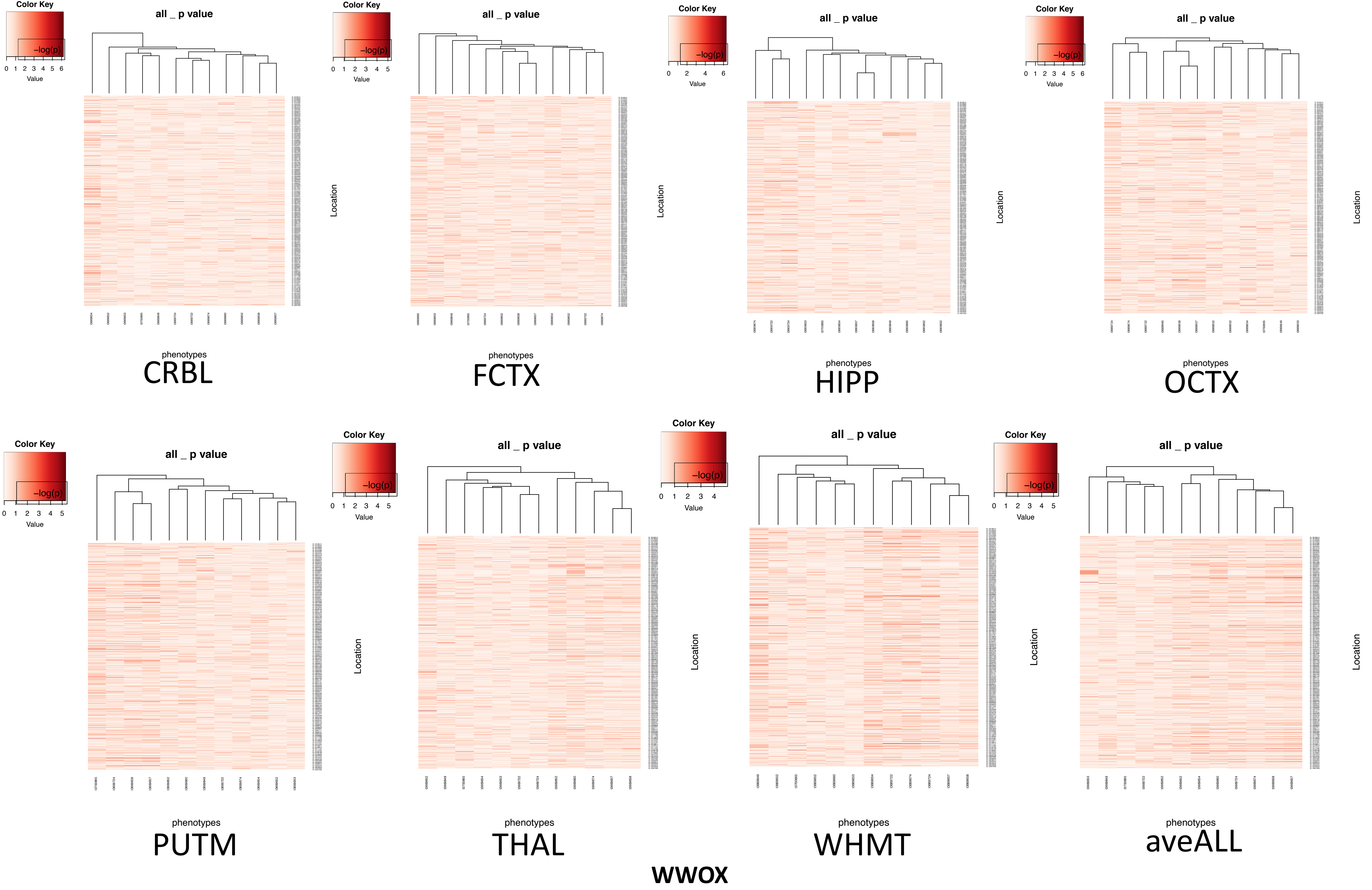

Supplementary figure 7. LRR-QTL in WWOX. Figure showing association results for log-R ratio with exon level gene expression in for different regions of brain in the UKBEC omni dataset. The MultiPhen method used was standard univariate model. This analysis was done on a gene-by-gene basis (see methods).

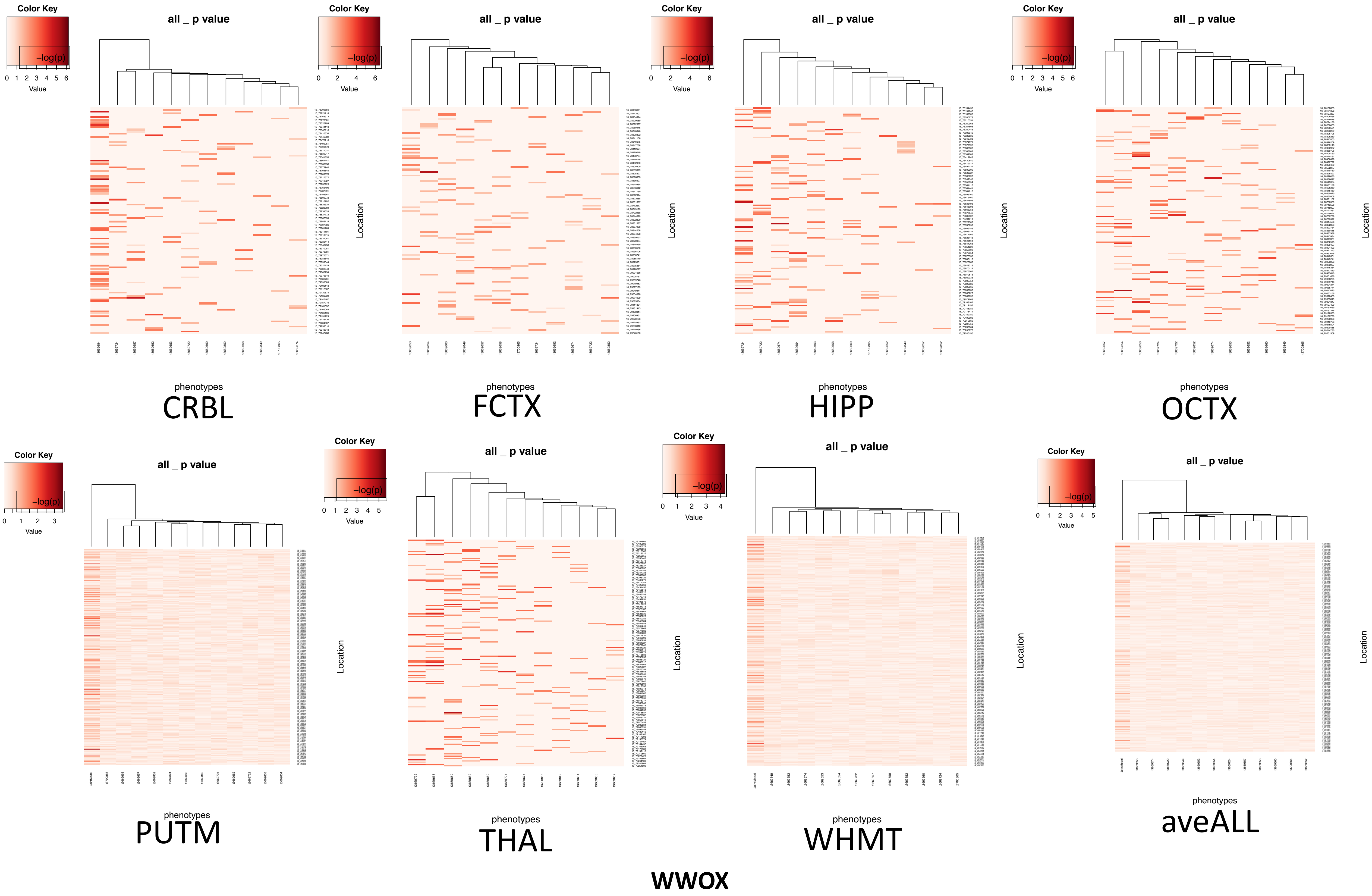

Supplementary figure 8. LRR-QTL in WWOX. Figure showing association results for log-R ratio with exon level gene expression in for different regions of the brain in the UKBEC dataset. The MultiPhen method used was joint model with variable selection. This analysis was done on a gene-by-gene basis (see methods).

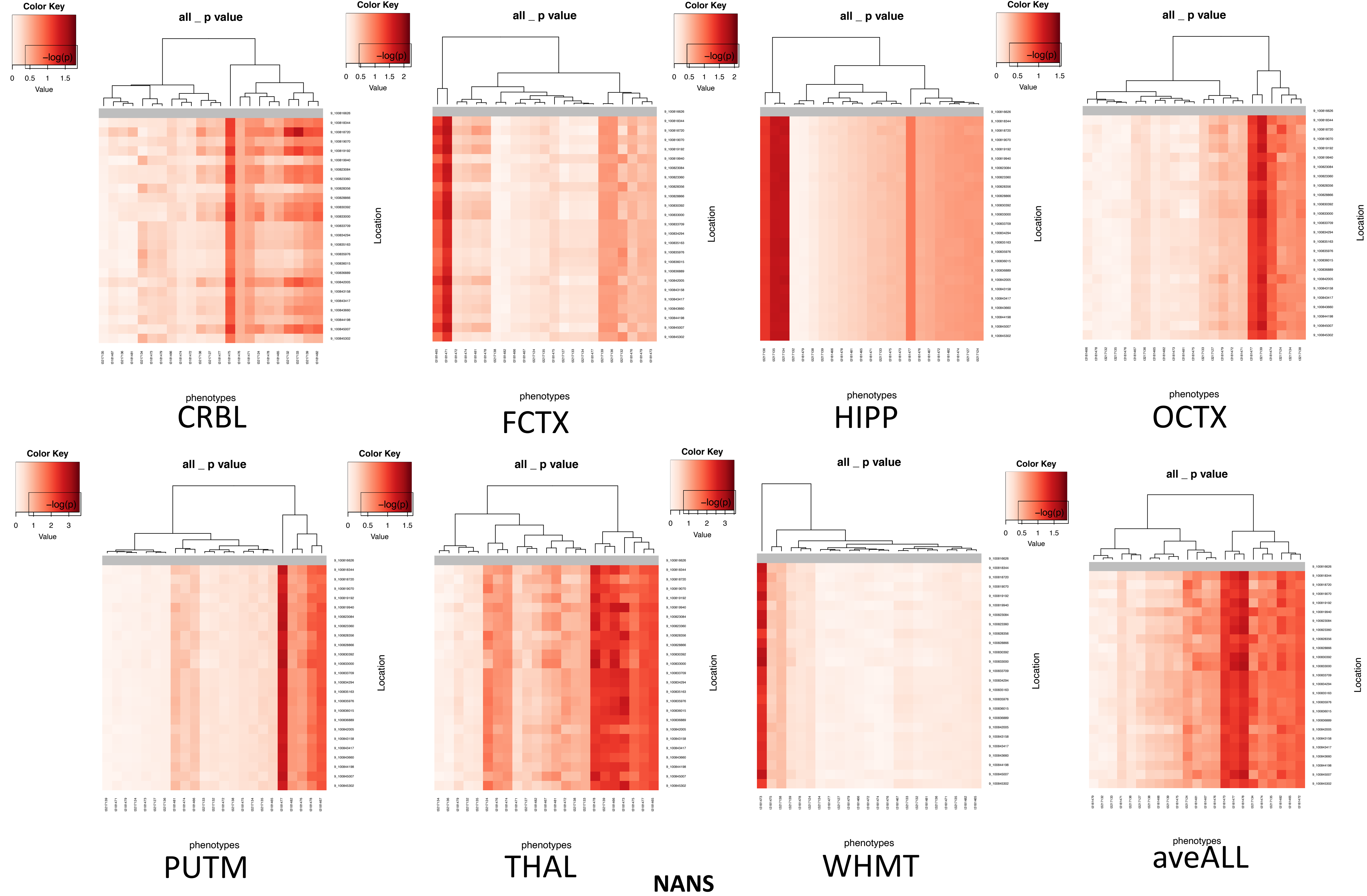

Supplementary figure 9. CNV-QTL in NANS. Figure showing association results for CNV genotypes with exon level gene expression in NANS for different regions of the brain in the UKBEC omni dataset. The MultiPhen method used was standard univariate model. Grey colour denotes missing data or NA. This analysis was done on a gene-by-gene basis (see methods).

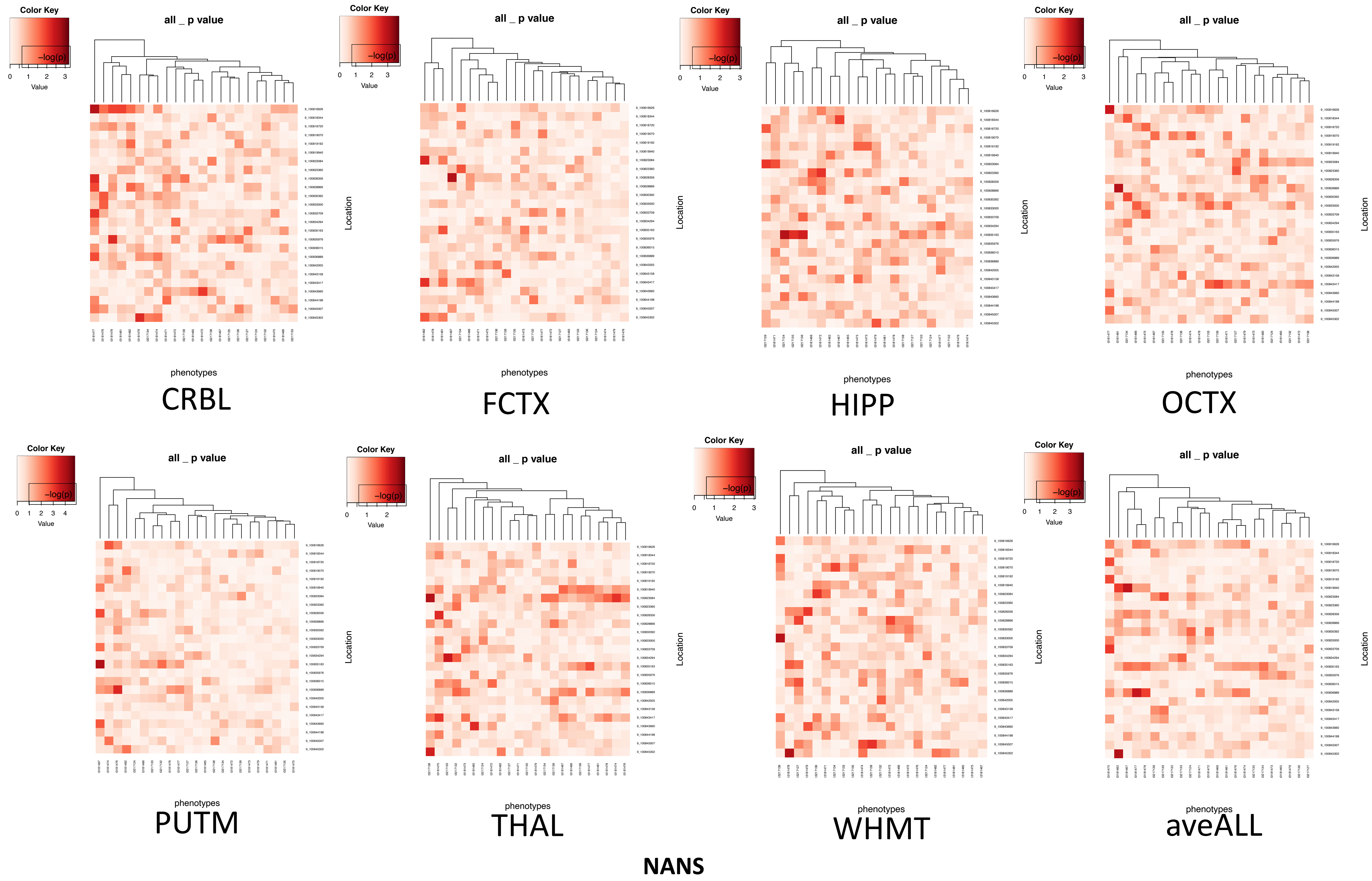

Supplementary figure 10. LRR-QTL in NANS. Figure showing association results for LRR with exon level gene expression in NANS for different regions of the brain in the UKBEC omni dataset. The MultiPhen method used was standard univariate model. This analysis was done on a gene-by-gene basis (see methods).

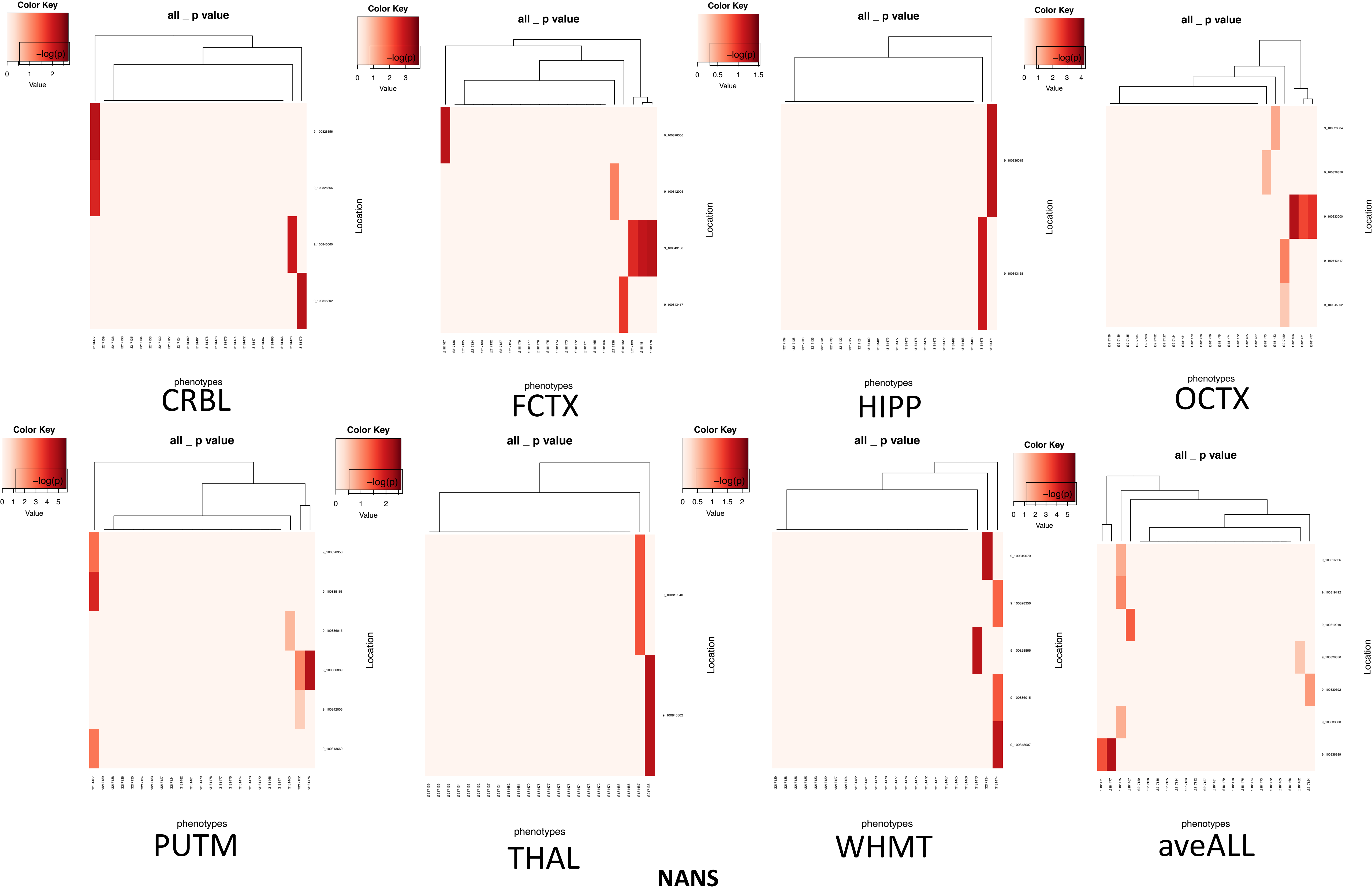

Supplementary figure 11. LRR-QTL in NANS. Figure showing association results for log-R ratio with exon level gene expression in NANS for different regions of the brain in the UKBEC omni dataset. The MultiPhen method used was joint model with variable selection. This analysis was done on a gene-by-gene basis (see methods).

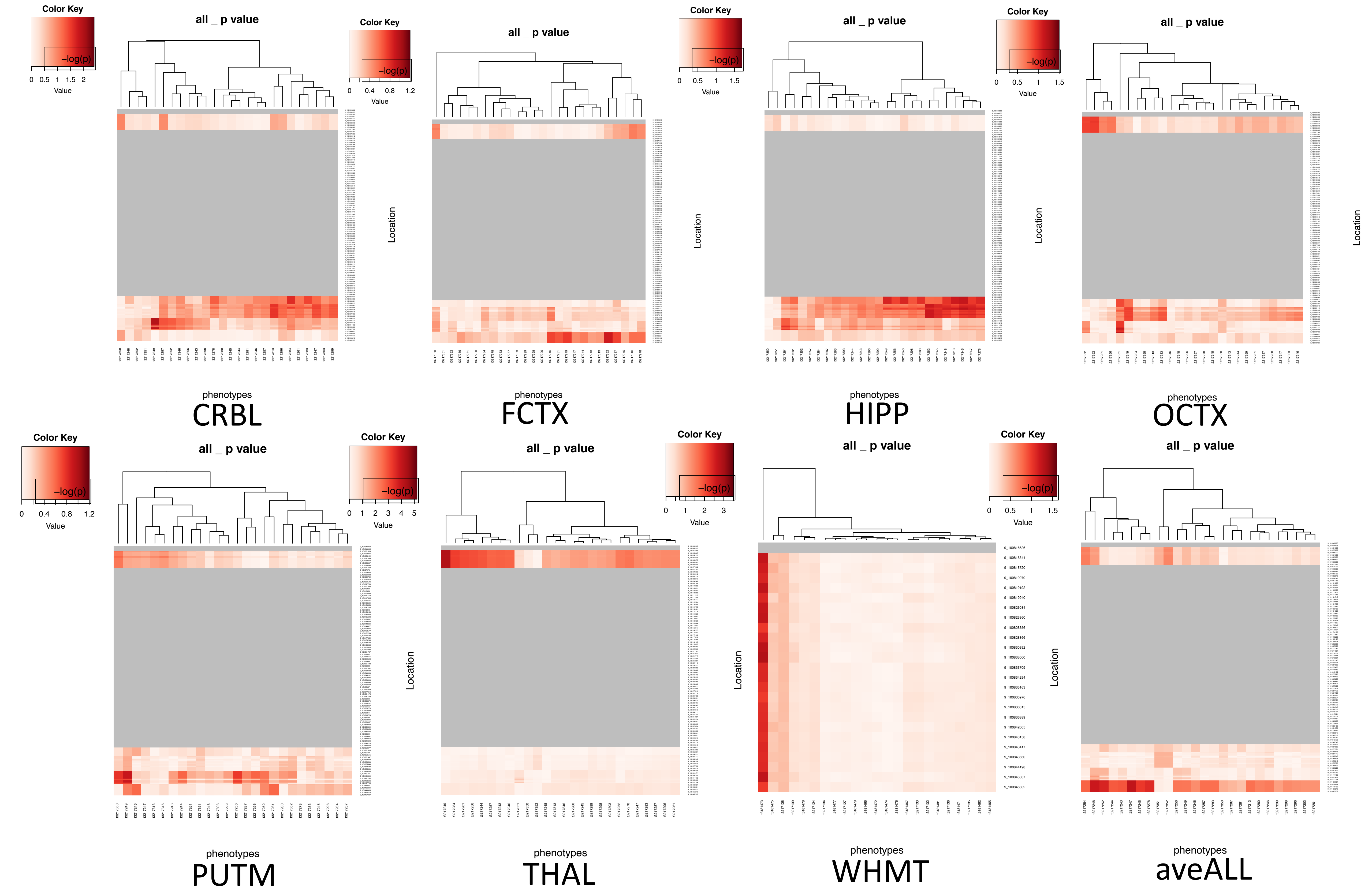

GABBR2

Supplementary figure 12. CNV-QTL in GABBR2. Figure showing association results for CNV genotypes with exon level gene expression in for different regions of the brain in the UKBEC dataset. The MultiPhen method used was standard univariate model. Grey colour denotes missing data or NA. This analysis was done on a gene-by-gene basis (see methods).

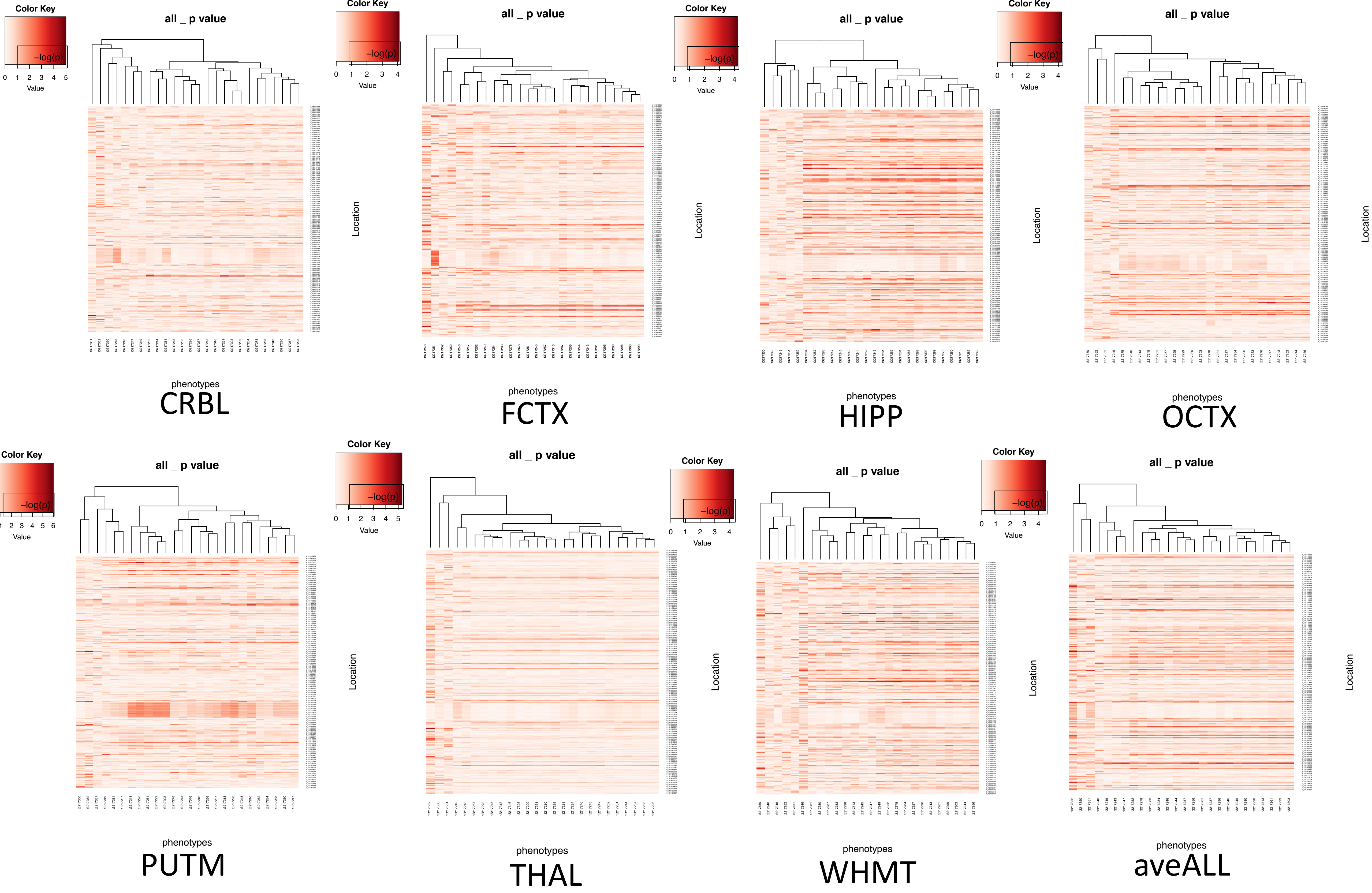

GABBR2

Supplementary figure 13. LRR-QTL in GABBR2. Figure showing association results for log-R ratio with exon level gene expression in for different regions of the brain in the UKBEC omni dataset. The MultiPhen method used was standard univariate model. This analysis was done on a gene-by-gene basis (see methods).

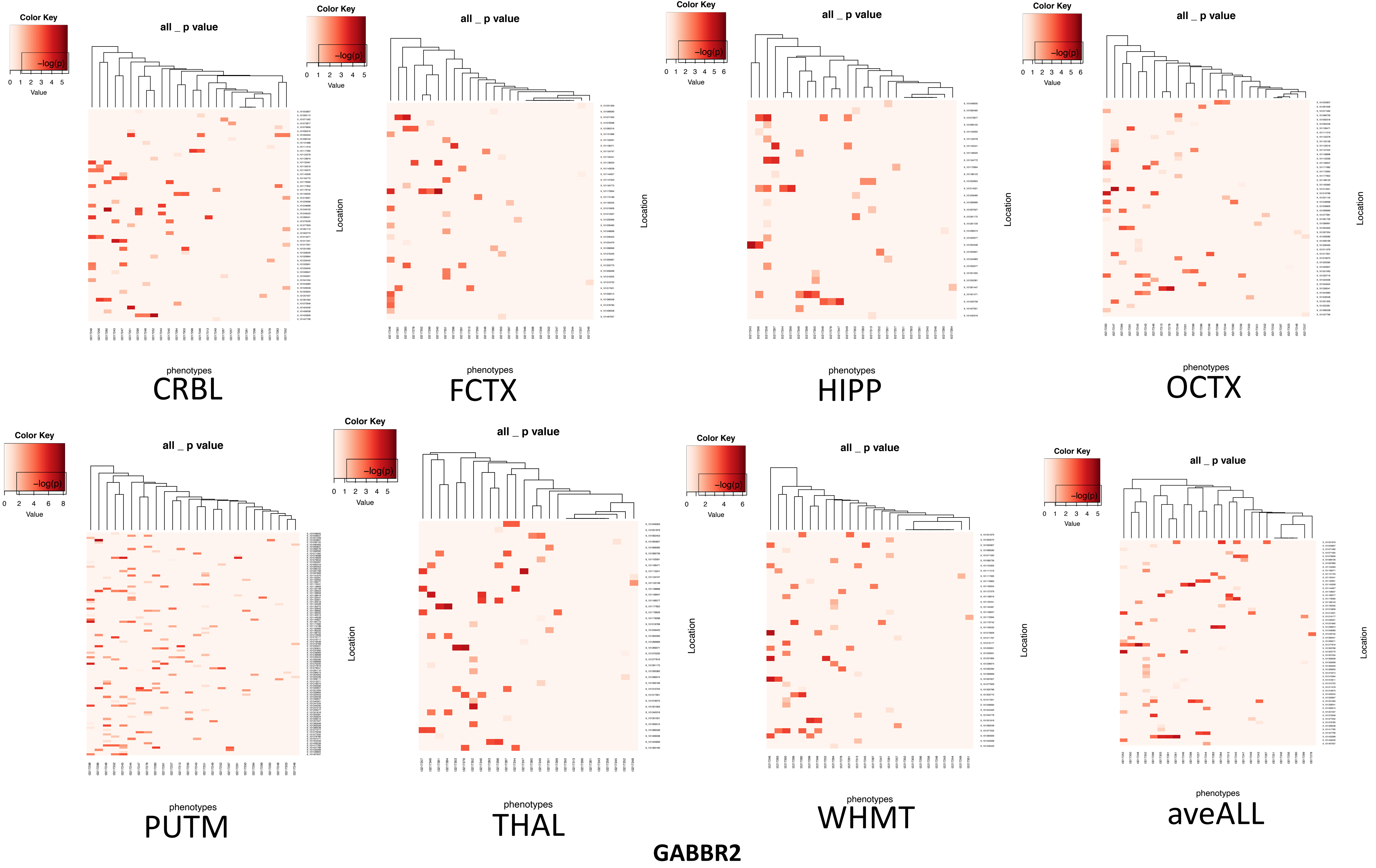

Supplementary figure 14. LRR-QTL in GABBR2. Figure showing association results for log-R ratio with exon level gene expression in for different regions of the brain in the UKBEC dataset. The MultiPhen method used was joint model with variable selection. This analysis was done on a gene-by-gene basis (see methods).

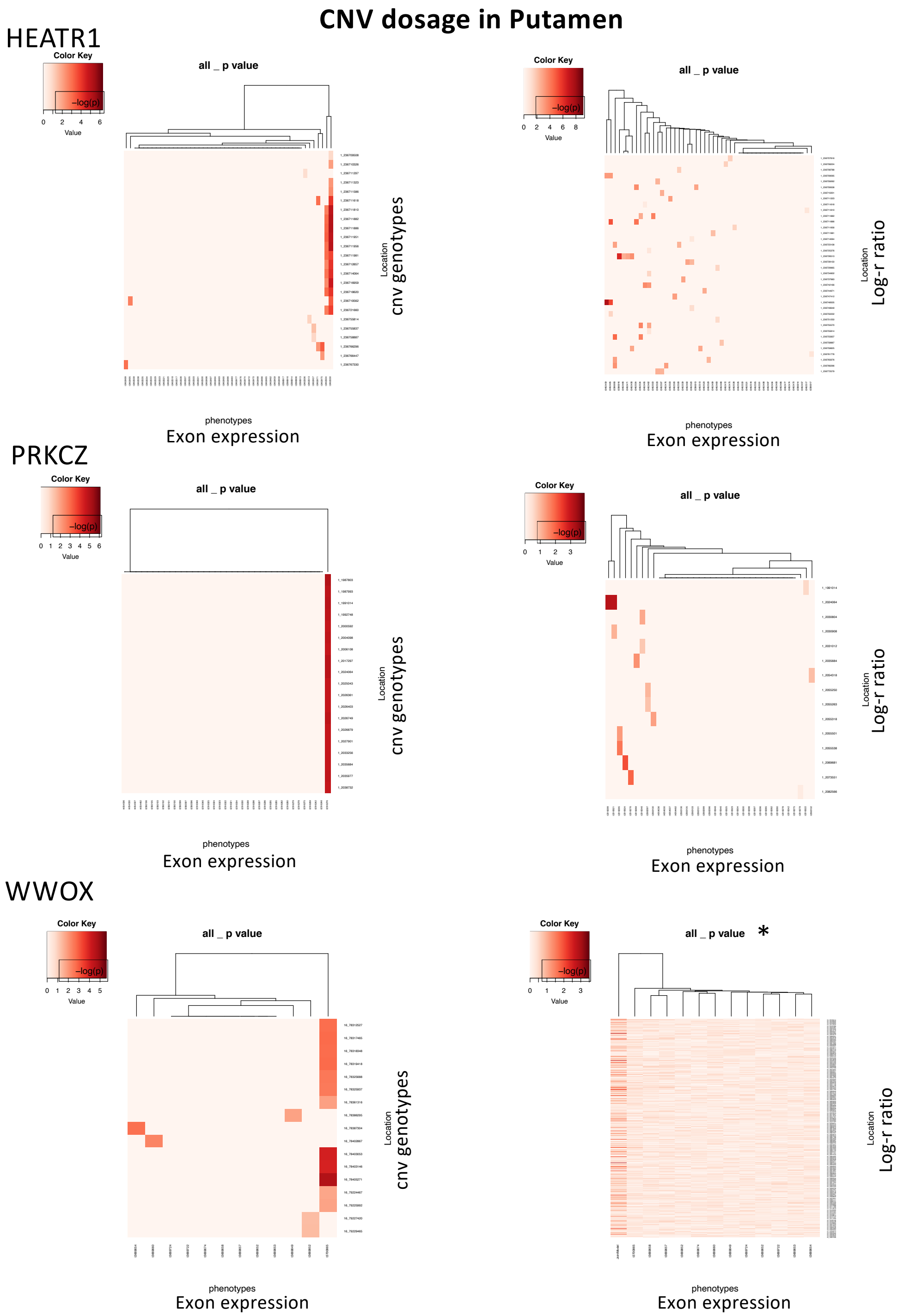

Supplementary figure 15. CNV-QTL in Putamen. Figure showing the association of CNV genotypes with exon level gene expression in three genes of interest namely HEATR1, PRKCZ and WWOX for the putamen brain region in the UKBEC dataset. The model of association used was MultiPhen joint model with variable selection for all panels except log-r-ratio result for WWOX (*). In this case the model was MultiPhen joint model only. This analysis was done on a gene-by-gene basis (see methods).

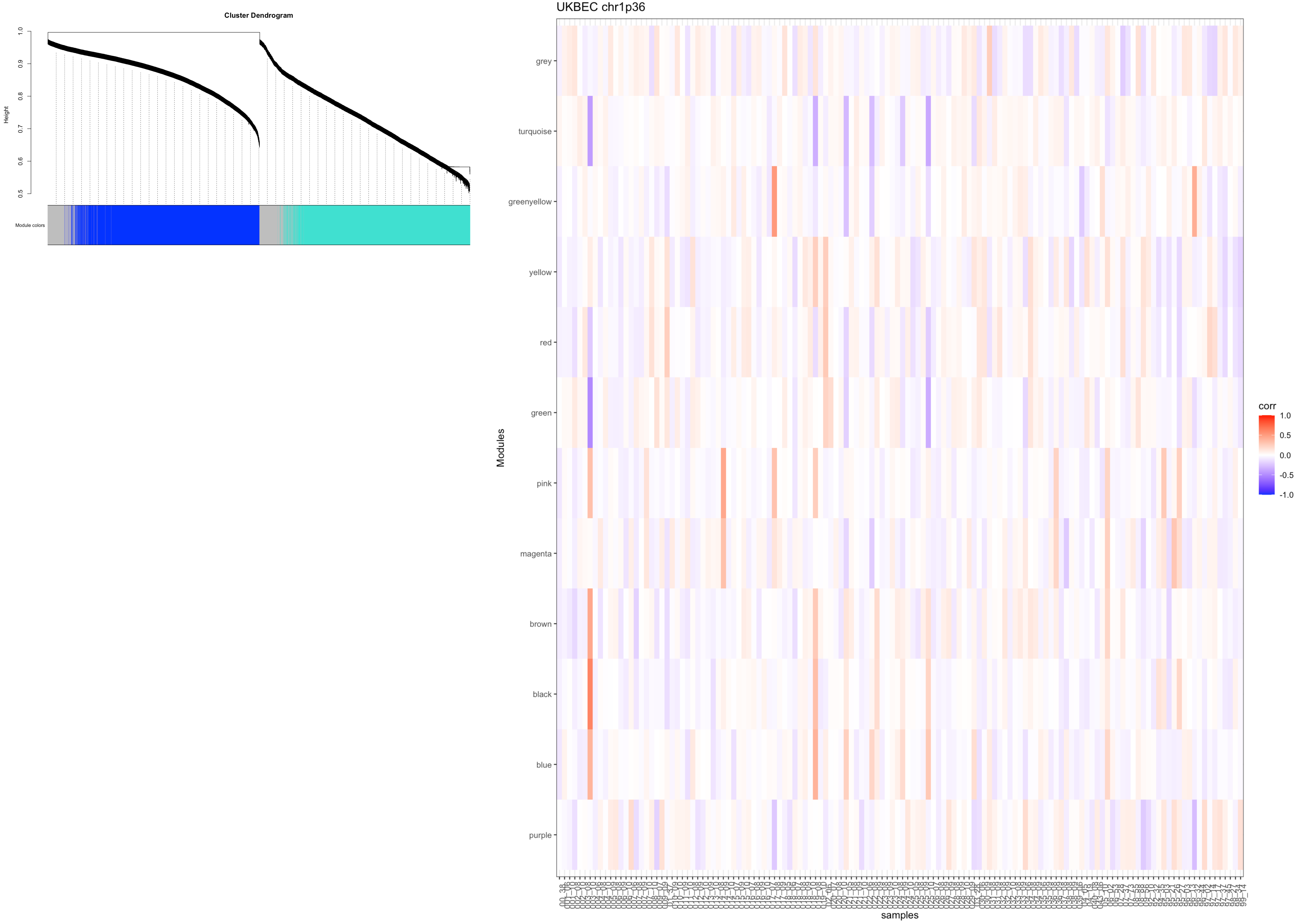

Supplementary figure 16. WGCNA analysis of the 1p36 region. Co-expression network analysis of the chromosome 1p36 region in the UKBEC dataset.

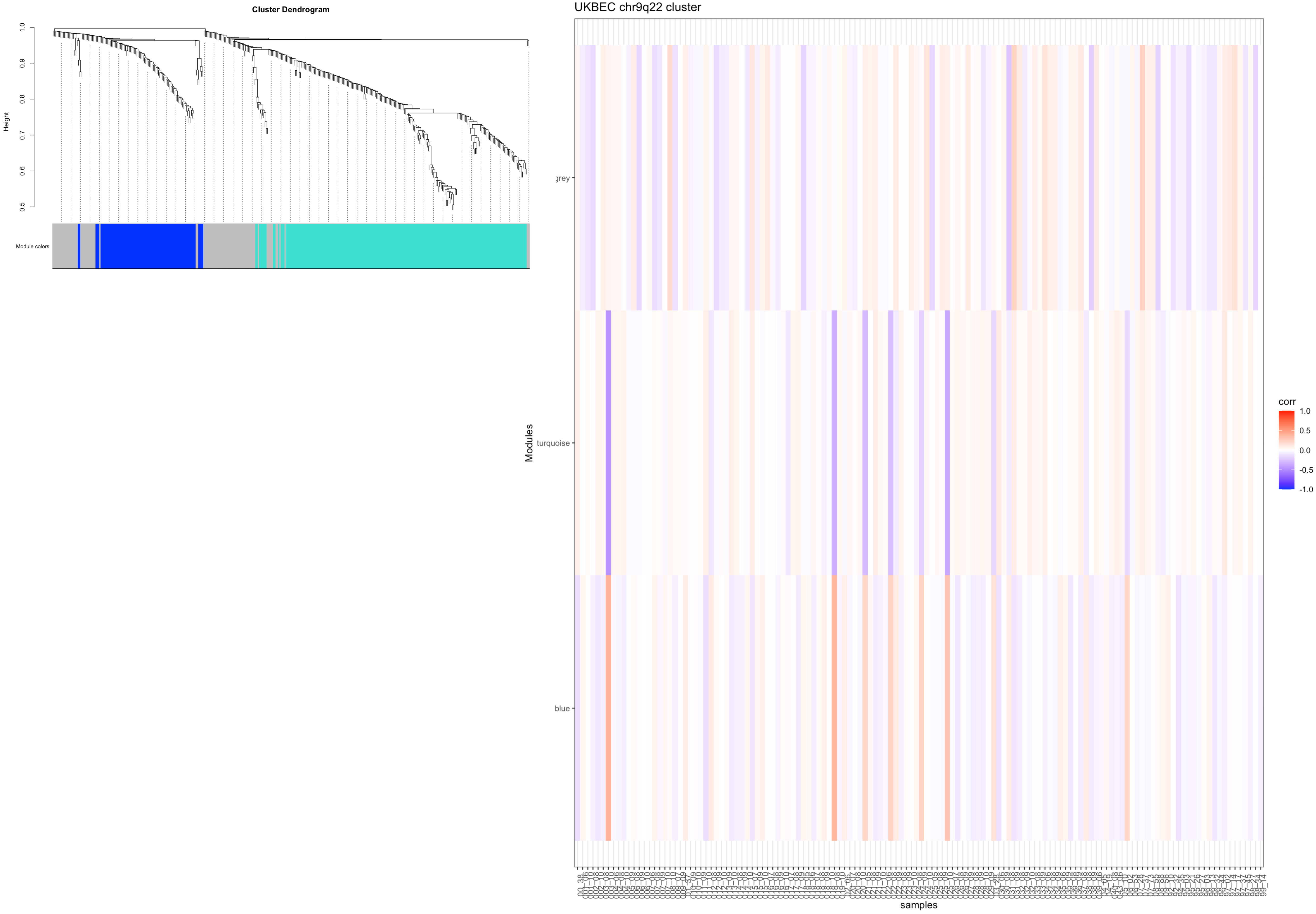

Supplementary figure 17. WGCNA analysis of the 9q22 region. Co-expression network analysis of the chromosome 9q22 region in the UKBEC dataset.

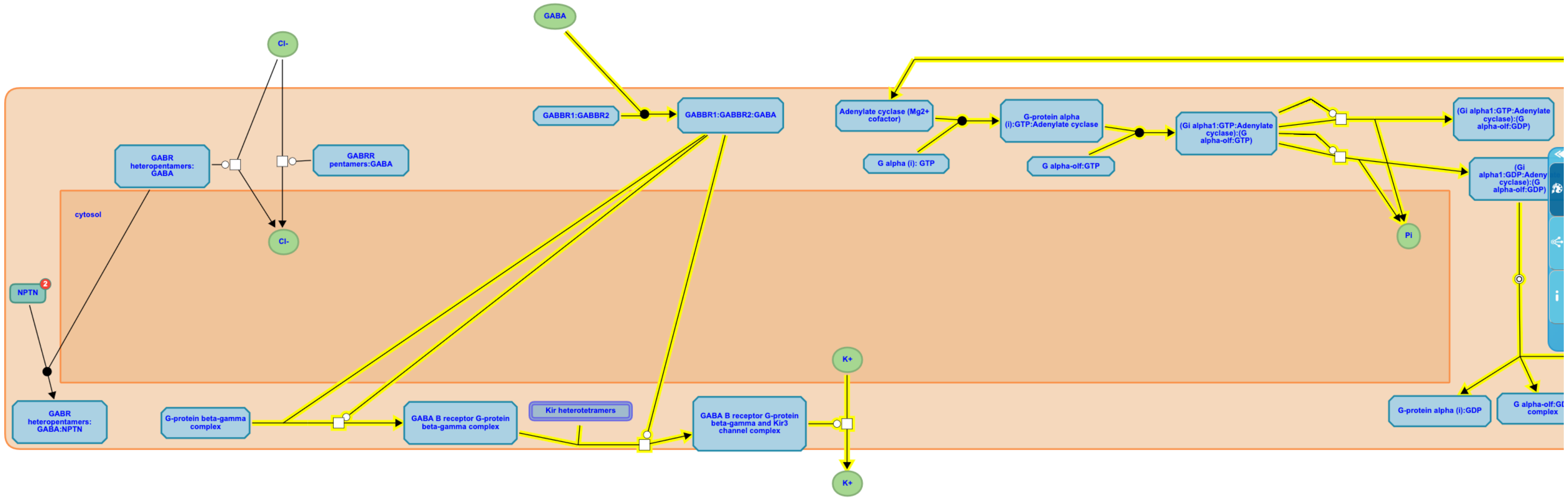

Supplementary figure 18. GABRB3 interaction with GNB1. Reactome pathway details showing GABA B receptors interacting with G protein alpha-I, 0 beta and gamma subunits which leads to the activation of GIRK (KIR3) potassium channels.

GRCh37 / hg19

WWOX WW domain containing oxidoreductase

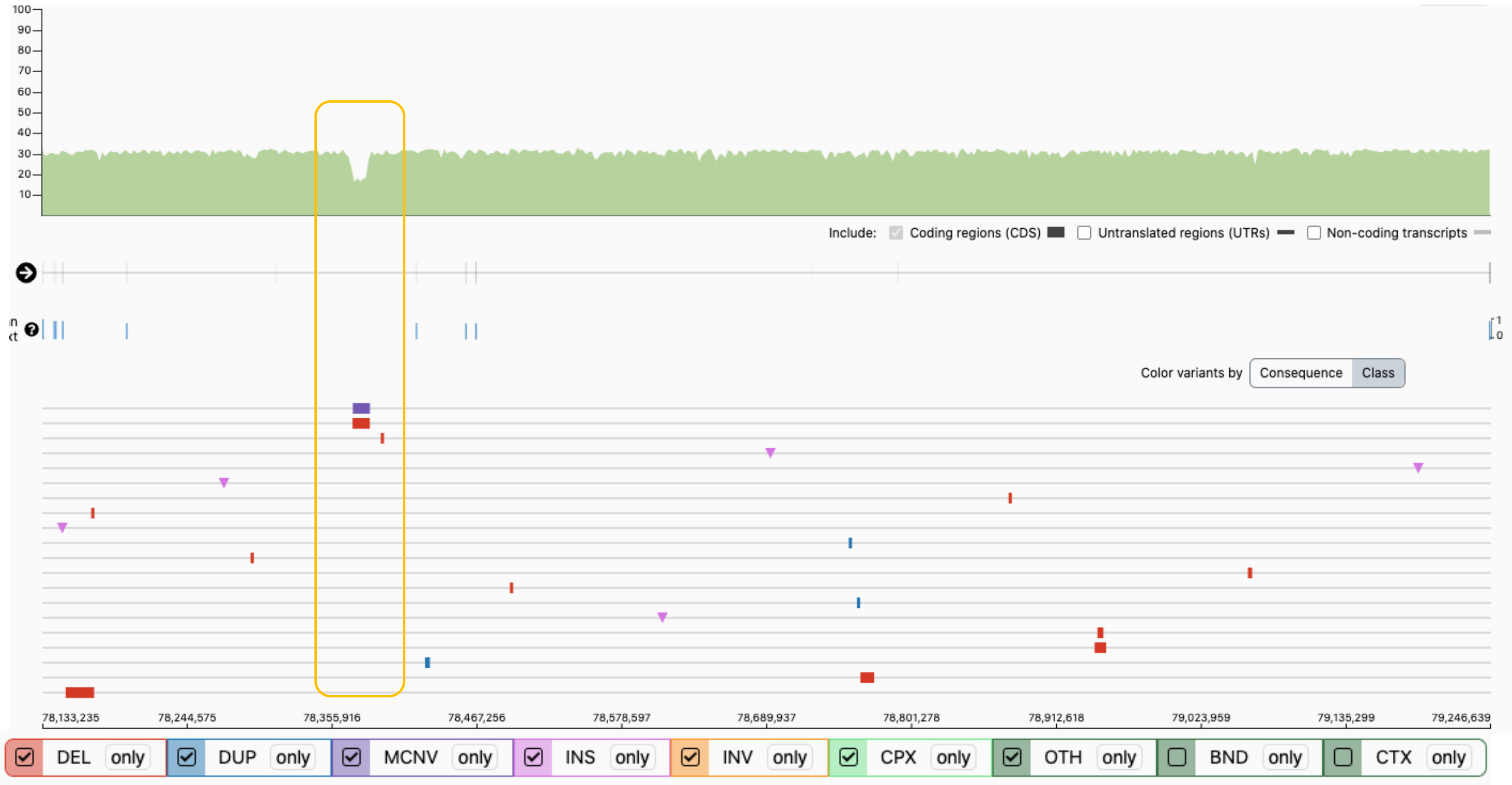

Variant ID	Source	Consequence	Class	Position	Size	Allele Count	Allele Number	Allele Frequency	Number of Homozygotes
MCNV_16_842	G	MCNV overlap	multi CNV	78371850 - 78385000	13.2 kb	5911	10847	5.45e-1	
DEL_16_156229	G	intronic	deletion	78371638 - 78384898	13.3 kb	7355	21694	3.39e-1	1447
DEL_16_156234	G	intronic	deletion	78394517 - 78394606	89 bp	6867	21572	3.18e-1	220
INS_16_101038	G	intronic	insertion	78692826	321 bp	4033	21640	1.86e-1	122
INS_16_101054	G	intronic	insertion	79190720	321 bp	2167	21506	1.01e-1	327
INS_16_101022	G	intronic	insertion	78272798	320 bp	1680	21394	7.85e-2	106
DEL_16_156287	G	intronic	deletion	78876652 - 78877577	925 bp	1165	21688	5.37e-2	93
DEL_16_156201	G	intronic	deletion	78171665 - 78172344	679 bp	994	21690	4.58e-2	41
INS_16_101020	G	intronic	insertion	78148585	280 bp	723	21434	3.37e-2	114
DUP_16_42640	G	partial duplication	duplication	78754211 - 78754269	58 bp	630	21366	2.95e-2	23
DEL_16_156215	G	intronic	deletion	78293346 - 78295653	2.31 kb	614	21692	2.83e-2	3
DEL_16_156307	G	intronic	deletion	79059786 - 79063002	3.22 kb	343	21694	1.58e-2	8
DEL_16_156247	G	intronic	deletion	78493716 - 78493942	226 bp	253	21674	1.17e-2	0
DUP_16_42642	G	partial duplication	duplication	78760484 - 78760563	79 bp	217	21520	1.01e-2	1
INS_16_101035	G	intronic	insertion	78609808	314 bp	218	21688	1.01e-2	43

Supplementary figure 19. CNVs in WWOX. Figure shows the CNVs in the WWOX gene from the gnomAD database (GRCh37 / hg19)

GRCh38 / hg38

WWOX WW domain containing oxidoreductase

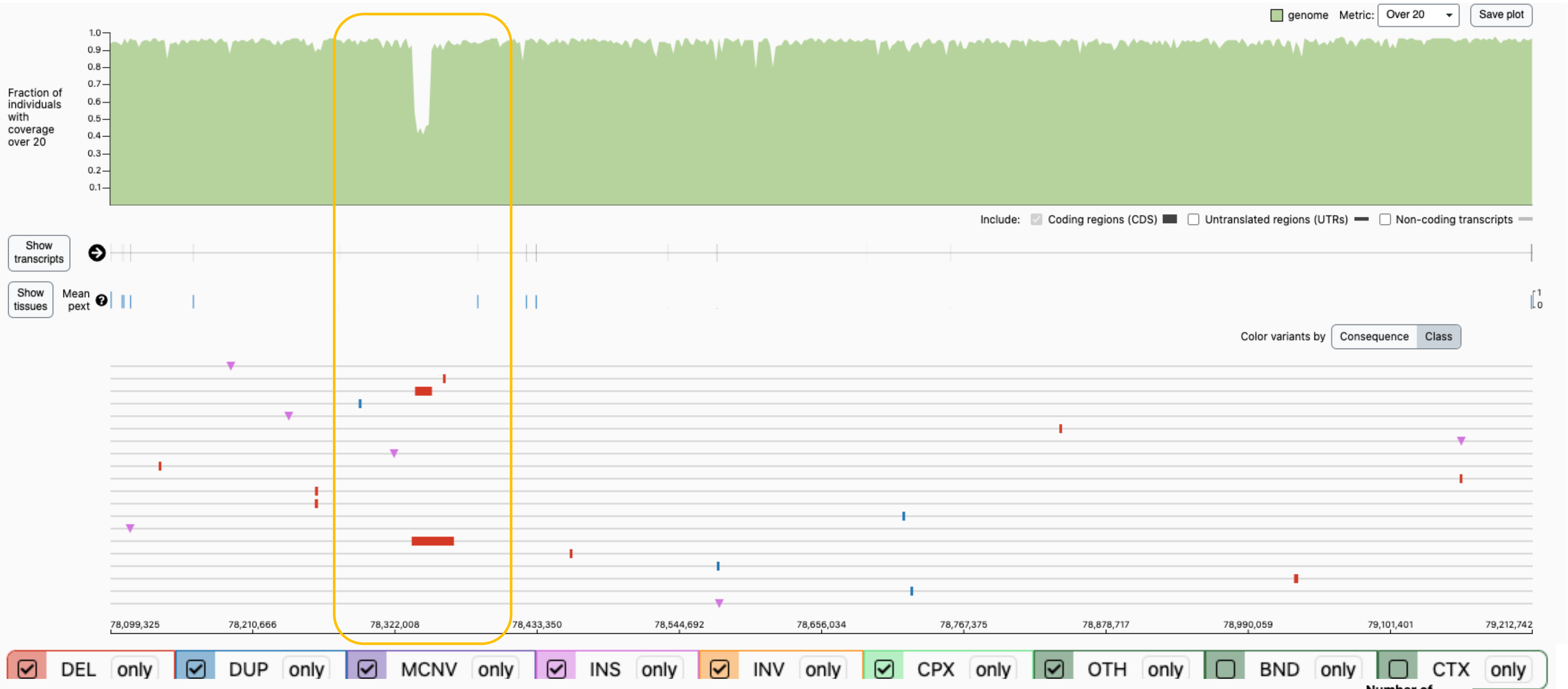

Variant ID	Source	Consequence	Class	Position	Size	Allele Count	Allele Number	Allele Frequency	Number of Homozygotes
INS_CHR16_9E189D...	G	intronic	insertion	78193550	414 bp	67637	126088	5.36e-1	9824
DEL_CHR16_CDE38...	G	intronic	deletion	78360599 - 78360709	110 bp	64894	126092	5.15e-1	14174
DEL_CHR16_93E35...	G	intronic	deletion	78337741 - 78351001	13.3 kb	48695	126092	3.86e-1	8760
DUP_CHR16_566B8...	G	intronic	duplication	78294719 - 78294777	58 bp	15043	126034	1.19e-1	1181
INS_CHR16_16A7319F	G	intronic	insertion	78238901	298 bp	12091	125956	9.60e-2	1214
DEL_CHR16_8EF1F6...	G	intronic	deletion	78842755 - 78843680	925 bp	9857	125834	7.83e-2	1718
INS_CHR16_C8223...	G	intronic	insertion	79156823	317 bp	9858	125998	7.82e-2	1296
INS_CHR16_6DF4B...	G	intronic	insertion	78321335	66 bp	8611	124644	6.91e-2	4
DEL_CHR16_B4BC8...	G	intronic	deletion	78137768 - 78138447	679 bp	7414	126080	5.88e-2	883
DEL_CHR16_481109...	G	intronic	deletion	79156451 - 79156823	372 bp	5093	119200	4.27e-2	56
DEL_CHR16_16669...	G	intronic	deletion	78259449 - 78261782	2.33 kb	3126	117920	2.65e-2	92
DEL_CHR16_7C36E...	G	intronic	deletion	78259283 - 78261752	2.47 kb	3083	117106	2.63e-2	45
DUP_CHR16_3F72E...	G	intronic	duplication	78720314 - 78720372	58 bp	3227	126092	2.56e-2	293
INS_CHR16_C64B8...	G	intronic	insertion	78114688	315 bp	3081	126092	2.44e-2	410
DEL_CHR16_EDE28...	G	intronic	deletion	78335124 - 78368198	33.1 kb	2285	126068	1.81e-2	1
DEL_CHR16_9E9BC...	G	intronic	deletion	78459819 - 78460045	226 bp	2002	125484	1.60e-2	61
DUP_CHR16_185D6...	G	intronic	duplication	78575007 - 78575095	88 bp	1766	117120	1.51e-2	460
DEL_CHR16_CA85E...	G	intronic	deletion	79025889 - 79029105	3.22 kb	1470	126088	1.17e-2	153
DUP_CHR16_2AB9D...	G	intronic	duplication	78726594 - 78726666	72 bp	1050	125638	8.36e-3	4
INS_CHR16_8AB01B...	G	intronic	insertion	78575911	314 bp	896	126010	7.11e-3	180

Supplementary figure 20. CNVs in WWOX. Figure shows the CNVs in the WWOX gene from the gnomAD database (GRCh38 / hg38)

Supplementary Table legends

Supplementary Table 1. Top results from all analyses. Table showing the top rank 1 results for SANAD, Australian cohort, meta-analysis, UKBEC and NABEC studies.

Supplementary Table 2. Clinvar database results for GNB1. Pathogenic CNV and SNP annotations for the GNB1 gene in the clinvar database.

Supplementary Table 3. Association results in SANAD for chromosome 1p36. Association results for seizure phenotypes in SANAD in the chromosome 1p36 region.

Supplementary Table 4. Association results for the WWOX gene. Association results for the drug-response phenotype in SANAD for CNV genotypes and LRR using univariate and multivariate models.

Supplementary Table 5. Methylation data for cancer cohorts. Whole-genome bisulfite sequencing data for different cancer types with read count >=700, namely: PBCA_DE, BOCA-FR and CLLE-ES (see methods for further details)

Supplementary Table 6. Methylation data for HFM1 gene. Next-generation bisulfite sequencing data for the HFM1 gene.

Supplementary Table 7. Copy number calls for cancer cohorts. Whole-genome CNV calls for PBCA-DE, BOCA-FR and CLLE-ES (see methods for further details)

Supplementary Table 8. Methylation data for the WWOX gene. Next-generation bisulfite sequencing data for WWOX gene in different cohorts.

Supplementary Table 9. NMF results for chromosome 1p36 in UKBEC. Frequency of exons in different gene programs uncovered through the NMF analysis of UKBEC dataset for NMF ranks 10 and 20 for gene expression matrices a) average all and b) full set.

Supplementary Table legends

Supplementary Table 10. WGCNA results for gene clusters in the UKBEC dataset. WGCNA based weighted co-expression network analysis for the chromosome 1p36 and 9q22 regions in the UKBEC study. The expression matrix used was the average expression across all brain regions (aveALL, see methods).

Supplementary Table 11. LRR results for SCN1B. Results for association of Log R Ratio with gene expression in different regions of the brain from the UKBEC dataset. Only three brain regions had p values < 1e-03 namely putamen, white matter and occipital cortex.

Supplementary Table 12. **Transcriptional sense of nearby genes.** Table showing the transcriptional sense and the NMF analysis (rank 2 to 6) of genes located close to each other in the genome. Top exon refers to the most frequent exon in the NMF gene programs. NMF analysis was done for every gene separately with a 5-kilo base window around the gene boundary (see methods).

Supplementary Table 13. Phenotype descriptions. Descriptions of epilepsy phenotypes in SANAD and Australian cohort.

Supplementary Table 14. cnvHap parameters. Parameters used for CNV calling through cnvHap.
